# Proteomic Profiling, Transcription Factor Modeling, and Genomics of Evolved Tolerant Strains Elucidate Mechanisms of Vanillin Toxicity in Escherichia coli

**DOI:** 10.1128/mSystems.00163-19

**Published:** 2019-06-11

**Authors:** Calum A. Pattrick, Joseph P. Webb, Jeffrey Green, Roy R. Chaudhuri, Mark O. Collins, David J. Kelly

**Affiliations:** a Department of Molecular Biology and Biotechnology, The University of Sheffield, Sheffield, United Kingdom; b Department of Biomedical Science, The University of Sheffield, Sheffield, United Kingdom; c biOMICS Biological Mass Spectrometry Facility, The University of Sheffield, Sheffield, United Kingdom; Northwestern University

**Keywords:** aldehyde, adaptive evolution, citrate synthase, copper, proteomics, stress responses

## Abstract

Vanillin (4-hydroxy-3-methoxybenzaldehyde) is an economically important flavor compound that can be made in bacterial cell factories, but toxicity is a major problem for cells producing this aromatic aldehyde. Using (i) a global proteomic analysis supported by multiple physiological experiments, mutant analyses, and inferred transcription factor modeling and (ii) adaptive laboratory evolution (ALE) of vanillin tolerance combined with genome-wide analysis of the underlying mutations, mechanisms of vanillin toxicity in Escherichia coli have been elucidated. We identified 147 proteins that exhibited a significant change in abundance in response to vanillin, giving the first detailed insight into the cellular response to this aldehyde. Vanillin caused accumulation of reactive oxygen species invoking adaptations coordinated by a MarA, OxyR, and SoxS regulatory network and increased RpoS/DksA-dependent gene expression. Differential fumarase C upregulation was found to prevent oxidative damage to FumA and FumB during growth with vanillin. Surprisingly, vanillin-dependent reduction pf copper (II) to copper (I) led to upregulation of the *copA* gene and growth in the presence of vanillin was shown to be hypersensitive to inhibition by copper ions. AcrD and AaeAB were identified as potential vanillin efflux systems. Vanillin-tolerant strains isolated by ALE had distinct nonsynonymous single nucleotide polymorphisms (SNPs) in *gltA* that led to increased citrate synthase activity. Strain-specific mutations in *cpdA*, *rob*, and *marC* were also present. One strain had a large (∼10-kb) deletion that included the *marRAB* region. Our data provide new understanding of bacterial vanillin toxicity and identify novel gene targets for future engineering of vanillin-tolerant strains of E. coli.

**IMPORTANCE** A particular problem for the biotechnological production of many of the valuable chemicals that we are now able to manufacture in bacterial cells is that these products often poison the cells producing them. Solutions to improve product yields or alleviate such toxicity using the techniques of modern molecular biology first require a detailed understanding of the mechanisms of product toxicity. Here we have studied the economically important flavor compound vanillin, an aromatic aldehyde that exerts significant toxic effects on bacterial cells. We used high-resolution protein abundance analysis as a starting point to determine which proteins are upregulated and which are downregulated by growth with vanillin, followed by gene expression and mutant studies to understand the mechanism of the response. In a second approach, we evolved bacterial strains with higher vanillin tolerance. Their genome sequences have yielded novel insights into vanillin tolerance that are complementary to the proteomics data set.

## Abstract (precis)

A particular problem for the biotechnological production of many of the valuable chemicals that we are now able to manufacture in bacterial cells is that these products often poison the cells producing them. Solutions to improve product yields or alleviate such toxicity using the techniques of modern molecular biology first require a detailed understanding of the mechanisms of product toxicity. Here we have studied the economically important flavor compound vanillin, an aromatic aldehyde that exerts significant toxic effects on bacterial cells. We used high-resolution protein abundance analysis as a starting point to determine which proteins are upregulated and which are downregulated by growth with vanillin, followed by gene expression and mutant studies to understand the mechanism of the response. In a second approach, we evolved bacterial strains with higher vanillin tolerance. Their genome sequences have yielded novel insights into vanillin tolerance that are complementary to the proteomics data set.

## INTRODUCTION

Vanillin (4-hydroxy-3-methoxybenzaldehyde) is the principal organoleptic component of vanilla flavoring and is traditionally sourced from the beans of the Vanilla planifolia plant. The widespread use of vanillin in food, beverages, cosmetics, and pharmaceuticals results in a huge demand (1, 2). However, less than 1% is now sourced naturally, with the rest produced largely through chemical synthesis. Vanillin was also produced in North America from the 1930s to 1991 by a hybrid fermentation/chemical process using lignin-containing waste resulting from wood pulping (3). With a greater consumer demand for “natural flavors,” there is a growing desire to produce vanillin by bioconversion from natural substrates. Furthermore, vanillin is a precursor for the production of polymers such as polyesters, phenolic resins, and thermosetting plastics (4). Therefore, the biotechnological exploitation of the microbial conversion of lignin-derived substrates such as eugenol, isoeugenol, and ferulic acid into vanillin is now a key area of commercial endeavor (5).

Current biotechnological approaches to vanillin synthesis involve either manipulating organisms that have the inherent ability to metabolize vanillin, such as *Rhodococcus* sp. (6), *Pseudomonas* sp. (7, 8), *Amycolatopsis* sp. (9), and *Streptomyces* sp. (10), or imbuing an alternative species with the capacity to produce it via heterologous enzyme expression. Vanillin is not an intermediate in metabolic pathways in a model organism such as Escherichia coli, which is used as a platform for vanillin production through heterologous expression of the required enzymes (11–13). Nevertheless, E. coli still harbors endogenous enzymes capable of the conversion of aldehydes into alcohols, such as aldo-keto reductases (DkgA, DkgB, and YeaE) and aldehyde dehydrogenases (YqhD, YahK, and YjgB) (14, 15). Deletion of multiple genes encoding these enzymes has been shown to result in increased accumulation of aromatic aldehydes (16).

A major problem with using bacterial cell factories for vanillin production is the toxicity of this compound. The electrophilic nature of an aldehyde functional group makes it highly reactive, and the damaging toxicity of aldehydes to a variety of cell types is well known (17–19). However, a comprehensive understanding of the mechanism of aldehyde toxicity at a physiological level in bacteria is yet to be established. Direct DNA damage and a detrimental NADPH drain (16, 20–22) have been identified as common effects of aldehydes on bacteria, while evidence of oxidative stress was found in yeast (23). A previous study concluded that vanillin has a bacteriostatic effect as a result of being a hydrophobic (Log *P* of 1.21) membrane-active compound (24). A proteomics-based study analyzed the response to growth on vanillin of Pseudomonas putida KT2240 (25). Unlike E. coli, P. putida possesses a catabolic pathway where vanillin is an intermediate (8). In addition to changes in carbon metabolism, these proteomics data also showed an increase in oxidative stress defenses (catalases), chaperones (GroEL, GrpE, DnaK), low-molecular-mass-compound synthesis (trehalose, proline, and betaine accumulation), and the presence of a wide range of efflux pumps that have been shown to be involved in solvent tolerance (25).

In this study, we investigated the response to and mechanism of toxicity of vanillin in E. coli BW25113 using a quantitative label-free proteomics workflow as a starting point. E. coli BW25113 is a well-characterized strain of E. coli from which the Keio collection of in-frame, single-gene knockouts was created (26) and has been engineered for production of chemicals as reported previously (27, 28). The results of the proteomics analyses were modeled to infer transcription factor (TF) activity and used along with extensive mutant studies to investigate mechanisms of toxicity and to identify targets that might be capable of being manipulated to improve vanillin tolerance. In a second approach, four strains were evolved from BW25113 by repeated subculturing in the presence of increasing concentrations of vanillin. Their genome sequences provide further insight into the physiological basis of vanillin toxicity and tolerance.

## RESULTS AND DISCUSSION

### Vanillin-dependent global changes in protein abundance assessed by high-resolution LC-MS/MS.

**FIG 1:**
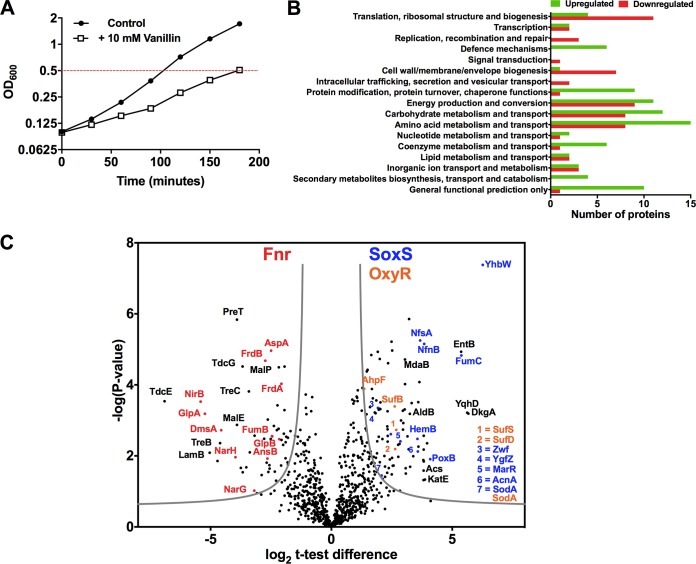
Summary of proteome changes in E. coli BW25113 in response to vanillin. (A) Cells from four independent replicate cultures grown without or with 10 mM vanillin were harvested for proteomic analysis at the same cell density (OD_600_ of 0.5) represented by the red dotted line in the growth curves shown. (B) Histogram of numbers of upregulated (green) and downregulated (red) proteins during growth with vanillin identified by LC-MS/MS, classified according to COG/NCBI functional categories. Five proteins that were upregulated, NfnB, HdhA, YghA, YbjP, and YbgI, do not have an assigned COG; one protein that was downregulated, YjjL, is also unassigned. (C) Volcano plot of the *P* values versus the log2 *t* test differences between the cultures grown without or with 10 mM vanillin. From the total data set of proteins identified, 147 proteins were significantly changed in abundance; the results of *t* tests of the quantitative data are shown here. Proteins indicated above the gray lines showed a significant change in abundance during growth with vanillin. A selection of proteins belonging to the Fnr regulon (red), the SoxS regulon (blue), and the OxyR regulon (orange) is shown. A selection of additional proteins of interest (black) is shown. Full lists of proteins showing significant changes are given in Tables 1 and 2.

Four independent cultures of E. coli BW25113 were grown aerobically in lysogeny broth (LB) in the absence or presence of a concentration of vanillin (10 mM) that slowed growth but was not completely inhibitory (a typical growth curve is shown in Fig. 1A). We used complex media in this work, in keeping with previous publications on vanillin production in E. coli (11, 29) and because Ni et al. (30) showed that LB was superior to minimal M9 minimal medium for vanillin production. Cell-free extracts were prepared from cells that had reached mid-exponential phase in each case and processed for mass spectrometry (MS) analysis. A label-free quantitative proteomics approach that included one-dimensional (1-D) SDS-PAGE and liquid chromatography-tandem mass spectrometry (LC-MS/MS) analysis was used to determine protein expression differences in response to growth with or without vanillin. We identified 1,885 proteins (1% false-discovery rate [FDR]), corresponding to 46% coverage of the E. coli BW25113 proteome (see Table S2 in the supplemental material).

Label-free quantification (LFQ) was performed using MaxQuant LFQ intensities, and statistical analysis was performed using Perseus (31) as follows. The data set was filtered to remove proteins with fewer than three valid LFQ values in at least one group, leading to accumulation of a set of 850 quantified proteins for statistical analysis (Table S3). LFQ intensities were subjected to log2 transformation, and missing values were imputed using a downshifted normal distribution (width, 0.3; downshift, 1.8); pairwise comparisons of LFQ intensities for each replicate are shown in Fig. S1 in the supplemental material. In order to identify changes in protein expression, *t* tests of the quantitative data were performed with correction for multiple-hypothesis testing using a permutation-based FDR of 0.05 and an S0 = 1.

10.1128/mSystems.00163-19.1 FIG S1 Correlation plots of proteomic data replicates. Label-free quantification (LFQ) intensities for each protein identified in each replicate sample are plotted against each other. Download FIG S1, TIF file, 1.0 MB. Copyright © 2019 Pattrick et al. 2019 Pattrick et al. This content is distributed under the terms of the Creative Commons Attribution 4.0 International license.

In total, 147 proteins exhibited a significant change in abundance in response to vanillin, 93 proteins showed a ≥2.5-fold increase, and 54 showed a ≥2.5-fold decrease (all these proteins are listed with fold changes in Tables 1 and 2). These sets of differentially regulated proteins included both cytoplasmic and membrane proteins with a range of biological functions. Functional classification was performed according to the Cluster of Orthologous Groups (COG) database (NCBI) for both sets (Fig. 1B), which showed that a large proportion of the differentially regulated proteins were involved in amino acid and carbohydrate metabolism and in energy production. The largest differences between the upregulated and downregulated protein groups were in the “defense mechanisms” and “posttranslational modification, protein turnover, chaperone functions” classifications, suggesting that vanillin was eliciting a cellular stress adaptation response. This was underlined by evidence indicating that multiple members of specific regulons involved with stress adaptation, for example, the SoxS, OxyR, and Fnr regulons, showed correlated changes in abundance (Fig. 1C). Consistent with the slower growth seen in the presence of vanillin, there was also a pattern of downregulation of proteins involved in “translation, ribosomal structure, and biogenesis,” “replication, recombination, and repair,” and “cell wall/membrane/envelope biogenesis,” indicating a reprogramming of metabolism away from growth and reproduction. The proteomic analysis identified many proteins related by membership in the same regulon or by being present in the same operon, with similar changes in abundance (Fig. 1C; see also Tables 1 and 2), suggesting that this approach reliably reports the effects of vanillin on the proteome. In addition, reverse transcription-PTR (RT-PCR) analysis of selected genes (see below) showed vanillin-dependent changes similar to the changes in the cognate protein abundance, further validating the proteomic data.

**TABLE 1:** Proteins quantified by label-free proteomic analysis that showed a significant increase in abundance in response to vanillin treatment^*a*^

Protein name	Protein description	Fold increase in abundance	*P* value
Oxidative stress defense			
AhpC	Alkyl hydroperoxide reductase subunit C	4.1	1.86E−05
AhpF	Alkyl hydroperoxide reductase subunit F	2.6	1.30E−04
KatE	Catalase HPII	4.8	4.65E−02
MarR	Multiple antibiotic resistance protein	9.4	3.89E−03
OsmC	Peroxiredoxin	4.4	2.74E−03
SodA	Superoxide dismutase (Mn)	5.0	3.58E−02

Glutathione			
GrxB	Glutaredoxin-2	7.5	4.94E−04
GshB	Glutathione synthetase	8.4	1.44E−02
Gss	Glutathionylspermidine synthetase/amidase	3.4	5.99E−04
GstA	Glutathione *S*-transferase	4.2	1.14E−02
YghU	Disulfide-bond oxidoreductase	6.5	1.01E−03
YqjG	Glutathionyl-hydroquinone reductase	3.7	5.01E−04

Low-MW compound accumulation			
GadC	Glutamate/γ-aminobutyrate antiporter	5.5	4.87E−03
GltB	Glutamate synthase (NADPH) large chain	2.8	9.58E−02
OtsA	α,α-Trehalose-phosphate synthase (UDP forming)	4.6	1.48E−03

Chaperones and proteases			
CbpA	Curved DNA-binding protein	3.4	3.89E−03
ClpB	Chaperone protein	2.8	3.78E−05
Dcp	Periplasmic serine endoprotease	3.1	1.44E−05
DegP	Periplasmic serine endoprotease	5.7	1.08E−05
HchA	Molecular chaperone Hsp31 and glyoxalase 3	9.6	2.72E−03
HslO	33-kDa chaperonin	4.3	2.23E−02
PepB	Peptidase B	5.2	2.45E−05

Metal homeostasis			
EntB	Enterobactin synthase component B	37.2	1.17E−05
EntE	Enterobactin synthase component E	8.5	1.19E−03
EntF	Enterobactin synthase component F	23.1	6.64E−03
MoaB	Molybdenum cofactor biosynthesis protein B	9.2	1.40E−06
SufB	FeS cluster assembly protein	5.9	4.04E−04
SufD	FeS cluster assembly protein	5.2	6.42E−03
SufS	Cysteine desulfurase	5.6	1.86E−03

Aldehyde detoxification			
Ahr	Aldehyde reductase	8.5	1.82E−03
AldB	Aldehyde dehydrogenase B	7.9	6.58E−04
DkgA	2,5-Diketo-d-gluconic acid reductase A	41.1	6.46E−04
YahK	Aldehyde reductase	3.6	3.90E−02
YghA	Uncharacterized oxidoreductase	4.3	1.87E−02
YqhD	Alcohol dehydrogenase	34.8	6.11E−04

Central carbon metabolism and energy production			
AceA	Isocitrate lyase	5.1	3.15E−02
AceB	Malate synthase A	6.4	6.52E−03
AcnA	Aconitate hydratase 1	8.0	7.53E−03
Acs	Acetyl-coenzyme A synthetase	5.6	2.60E−02
FbaB	Fructose-bisphosphate aldolase class 1	6.9	2.82E−03
FumC	Fumarate hydratase class II	38.4	1.49E−05
GltA	Citrate synthase	3.3	1.51E−02
Mdh	Malate dehydrogenase	4.0	3.12E−04
MtlA	PTS mannitol-specific EIICBA component	4.1	4.68E−04
PfkB	ATP-dependent 6-phosphofructokinase isozyme 2	3.6	2.00E−02
Ppk	Polyphosphate kinase	3.5	6.31E−04
PoxB	Pyruvate oxidase (ubiquinone reducing)	10.9	1.24E−02
Sad	Succinate semialdehyde dehydrogenase	6.8	1.02E−02
TalA	Transaldolase A	4.7	1.06E−02
TktB	Transketolase 2	5.3	2.03E−02
Zwf	Glucose-6-phosphate 1-dehydrogenase	3.6	4.46E−04
Nitrocompound detoxification			
NfsA	Oxygen-insensitive NADPH nitroreductase	12.8	5.64E−06
NfnB	Oxygen-insensitive NAD(P)H nitroreductase	14.8	7.03E−06

Amino acid biosynthesis			
AstC	Succinylornithine transaminase	4.1	4.80E−02
HisB	Histidine biosynthesis bifunctional protein	5.5	4.59E−03
HisC	Histidinol-phosphate aminotransferase	5.2	2.21E−02
HisD	Histidinol dehydrogenase	3.8	2.90E−03
HisG	ATP phosphoribosyltransferase	8.0	9.55E−05
IlvB	Acetolactate synthase isozyme 1 large subunit	3.9	3.32E−02
LysC	Lysine-sensitive aspartokinase 3	3.0	4.23E−04
PheA	P-protein	3.4	1.13E−02
TrpB	Tryptophan synthase beta chain	2.7	6.73E−04
YecD	Isochorismatase family protein	7.2	5.35E−04

Folate metabolism			
FolE	GTP cyclohydrolase 1	3.1	1.31E−04
PanB	3-Methyl-2-oxobutanoate hydroxymethyltransferase	3.5	8.48E−03
YbgI	Putative GTP cyclohydrolase 1 type 2	3.1	7.58E−03
YgfZ	tRNA-modifying protein	4.9	2.44E−03

β-d-Glucuronide and d-glucuronate degradation			
UidA	Beta-glucuronidase	5.6	1.78E−03
UxaC	Uronate isomerase	12.8	8.36E−05
UxuA	Mannonate dehydratase	4.1	1.00E−02

Uncharacterized			
YbjP	Uncharacterized lipoprotein	4.0	4.46E−02
YdhJ	Uncharacterized protein	3.5	1.06E−02
YeiR	Uncharacterized protein	6.4	1.90E−02
YhbW	Uncharacterized protein	77.1	4.18E−08
YjhC	Uncharacterized oxidoreductase	23.8	4.43E−03
YncE	Uncharacterized protein	9.5	1.31E−02

Others			
Add	Adenosine deaminase	3.5	2.75E−04
Amn	AMP nucleosidase	5.5	6.10E−06
AnmK	Anhydro-N-acetylmuramic acid kinase	3.2	2.39E−04
Cfa	Cyclopropane-fatty-acyl-phospholipid synthase	4.2	4.81E−04
CodA	Cytosine deaminase	3.2	2.99E−02
CurA	NADPH-dependent curcumin reductase	4.1	3.97E−03
DcyD	d-Cysteine desulfhydrase	4.4	3.35E−03
EmrA	Multidrug export protein	4.5	1.26E−02
GhrA	Glyoxylate/hydroxypyruvate reductase A	4.8	5.56E−04
HdhA	7-Alpha-hydroxysteroid dehydrogenase	10.4	3.08E−04
HemB	Delta-aminolevulinic acid dehydratase	9.0	3.32E−03
LysU	Lysine-tRNA ligase, heat inducible	3.8	1.18E−05
MdaB	Modulator of drug activity B	8.4	1.93E−05
MetG	Methionine-tRNA ligase	2.7	4.19E−05
MnmA	tRNA-specific 2-thiouridylase	2.8	1.60E−02
TorA	Trimethylamine-N-oxide reductase 1	8.1	5.48E−03
YadG	Uncharacterized ABC transporter ATP-binding protein	8.9	3.26E−04

a Fold increase data were calculated from the average abundances of four independent vanillin-treated replicates relative to those of the four independent untreated replicates. PTS, phosphotransferase system.

**TABLE 2:** Proteins quantified by label-free proteomic analysis that showed a significant decrease in abundance in response to vanillin treatment^*a*^

Protein name	Protein description	Fold decrease	*P* value
Anaerobic response and FeS-cluster-containing proteins			
DmsA	Dimethyl sulfoxide reductase	21.5	1.90E−03
FrdA	Fumarate reductase flavoprotein subunit	4.3	9.34E−05
FrdB	Fumarate reductase iron-sulfur subunit	6.5	2.09E−05
FumB	Fumarate hydratase class I, anaerobic	4.8	2.79E−03
GlpA	Anaerobic glycerol-3-phosphate dehydrogenase subunit A	24.7	6.51E−04
GlpB	Anaerobic glycerol-3-phosphate dehydrogenase subunit B	4.6	3.39E−03
MiaB	tRNA-2-methylthio-N(6)-dimethylallyladenosine synthase	9.1	1.56E−02
NarG	Respiratory nitrate reductase 1 alpha chain	6.2	9.52E−02
NarH	Respiratory nitrate reductase 1 beta chain	11.4	1.09E−02
NirB	Nitrite reductase (NADH) large subunit	37.4	2.96E−04
PreT	NAD-dependent dihydropyrimidine dehydrogenase subunit	15.4	1.46E−06
SdaB	l-Serine dehydratase 2	31.1	1.13E−01
TdcG	l-Serine/threonine dehydratase	12.3	3.04E−05
Glycyl radical proteins			
GrcA	Autonomous glycyl radical cofactor	5.2	1.88E−03
TdcE	PFL-like enzyme (ketobutyrate formate lyase)	60.6	2.89E−04
Maltose and trehalose			
LamB	Maltoporin	16.5	8.15E−03
MalE	Maltose-binding periplasmic protein	15.0	1.34E−03
MalP	Maltodextrin phosphorylase	4.5	3.21E−05
MalQ	4-Alpha-glucanotransferase	5.5	1.44E−03
TreB	PTS trehalose-specific EIIBC component	36.2	4.38E−03
TreC	Trehalose-6-phosphate hydrolase	9.5	1.53E−04
Porins			
FadL	Long-chain fatty acid transport protein	10.4	7.97E−03
OmpF	Outer membrane protein F	11.5	2.18E−02
Tsx	Nucleoside-specific channel-forming protein	5.3	2.82E−03
Replication and cell division			
DeaD	ATP-dependent RNA helicase	13.9	1.40E−02
MnmG	tRNA uridine 5-carboxymethylaminomethyl modification	7.6	2.89E−02
MurA	UDP-N-acetylglucosamine 1-carboxyvinyltransferase	3.6	2.89E−03
ParE	DNA topoisomerase 4 subunit B	6.1	2.10E−02
RimO	Ribosomal protein S12 methylthiotransferase	3.1	1.52E−02
RplD	50S ribosomal protein L4	3.2	1.78E−02
RplP	50S ribosomal protein L16	4.3	2.28E−02
RplT	50S ribosomal protein L20	3.4	2.78E−02
RplU	50S ribosomal protein L21	3.3	4.12E−03
RplY	50S ribosomal protein L25	4.0	3.47E−02
RpmB	50S ribosomal protein L28	3.2	1.86E−02
TtcA	tRNA 2-thiocytidine biosynthesis protein	8.7	2.84E−02
LPS biosynthesis			
rfbB	dTDP-glucose 4,6-dehydratase 1	3.8	3.06E−05
RfbD	dTDP-4-dehydrorhamnose reductase	4.3	1.17E−02
Aspartate/asparagine catabolism			
AnsB	l-Asparaginase 2	7.8	1.19E−02
AsnA	Aspartate-ammonia ligase	7.1	3.30E−03
AspA	Aspartate ammonia-lyase	5.7	1.09E−05
Glyoxylate degradation			
GarD	d-Galactarate dehydratase	3.8	3.55E−03
GarR	2-Hydroxy-3-oxopropionate reductase	8.7	2.70E−03
Uncharacterized			
YdhQ	Uncharacterized protein YdhQ	5.5	9.51E−04
YgeV	Uncharacterized σ54-dependent transcriptional regulator	3.4	2.01E−03
YjjI	Uncharacterized protein YjjI	6.2	3.36E−03
Others			
FtnA	Bacterial nonheme ferritin	5.2	1.83E−02
GatZ	d-Tagatose-1,6-bisphosphate aldolase subunit	4.1	2.37E−03
GuaC	GMP reductase	4.7	1.72E−03
HupA	DNA-binding protein HU-alpha	4.1	4.90E−03
HypB	Hydrogenase isoenzymes nickel incorporation protein	7.1	5.01E−02
NanA	N-Acetylneuraminate lyase	3.4	3.08E−03
PepE	Peptidase E	3.9	5.16E−04
RpoS	RNA polymerase sigma factor	4.9	3.77E−04

a Fold decrease was calculated from the average abundance of four independent vanillin-treated replicates relative to that of the four independent untreated replicates. LPS, lipopolysaccharide; PFL, pyruvate formate lyase.

### Probabilistic modeling of the proteomic data to infer transcription factor activity.

**FIG 2:**
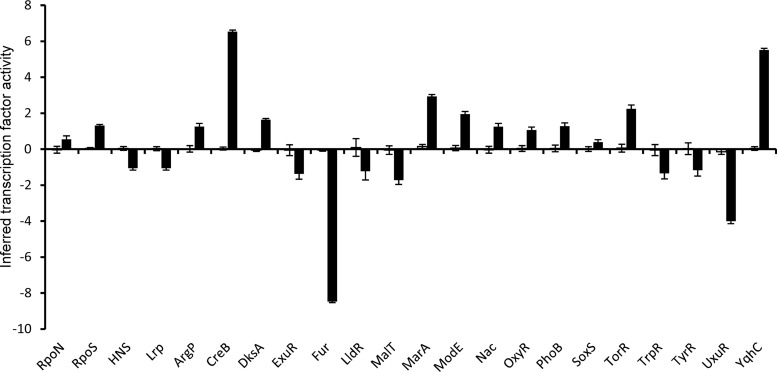
Inferred changes in transcription factor activities in response to vanillin. The output from the TFInfer program (Asif et al. [32]) in the absence (open bars) and presence (filled bars) of vanillin is shown. The error bars represent standard deviations provided by the posterior distributions. The full output is shown in Table S4.

**FIG 3:**
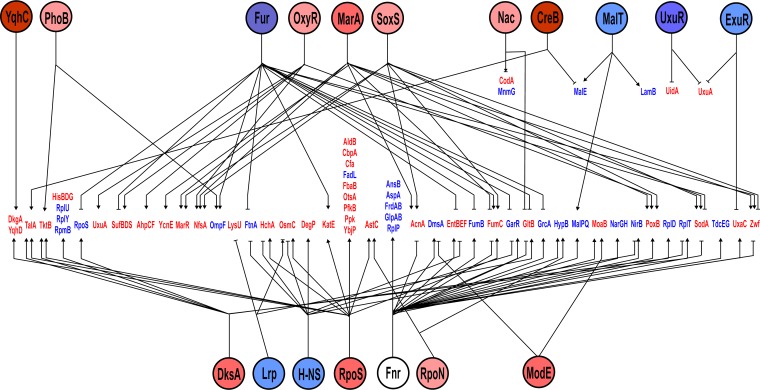
Major regulatory networks mediating changes in protein abundance in response to vanillin. The regulators are shown as named circles colored to indicated increased (red) or decreased (blue) activity in the presence of vanillin. The brightness of the shading is a visual indication of extent of the predicted changes in activities as depicted in Fig. 2; the brighter the color, the greater the response. The regulated proteins (red, increased abundance in the presence of vanillin; blue, decreased abundance) are aligned across the center of the diagram and are linked to the relevant regulators by as indicated by the following symbols: →, activation; –I, inhibition; →|, dual responses.

The proteomic data reported here necessarily include only those proteins that could be detected and identified. However, assuming that gene expression is ultimately correlated with the cellular protein complement, it should then be possible to obtain an unbiased indication of the changes in transcription factor (TF) activities that underpin the observed changes in protein abundance in response to vanillin. TFInfer was developed as a probabilistic modeling tool for genome-wide inference of TF activities from transcriptomic data (32). TFInfer is a state space model that combines a binary connectivity matrix that links genes to TFs with, in this case, changes in protein abundance. The model places Gaussian prior distributions over each TF activity and then applies a factorized variational approximation to infer the posterior distributions of TF activities that could account for the observed changes in gene expression. Although such models are simplifications, they provide insights into the mechanisms underpinning adaptive processes. TFs can be classified as global or local according to the breadth of their regulons, their capacity to interact with alternative sigma factors and coregulators, the number of “slave” TFs that they control, and the size of their evolutionary families (33). By these criteria, E. coli has seven global TFs (ArcA, Crp, Fis, Fnr, H-NS, IHF, and Lrp). In addition, E. coli employs the stress-responsive alternative sigma factors RpoE, RpoH, RpoN, and RpoS to redistribute RNA polymerase (34). The proteomic data set, consisting of 1,052 proteins detected in the absence and presence of vanillin, was simultaneously analyzed for changes in the activities of 123 TFs and of RpoE, RpoH, RpoN and RpoS. Two sigma factors, 2 global regulators, and 17 local regulators were predicted to exhibit altered activity (Fig. 2; see also Table S4). These regulators formed a complex network to coordinate the responses to the presence of vanillin (Fig. 3). The local regulator, YqhC, was strongly activated (Fig. 2), resulting in increased abundance of putative vanillin detoxification enzymes DkgA and YqhD (Table 1). A major subnetwork indicative of an oxidative stress response was formed by the activation of the local TFs MarA/MarR, OxyR, and SoxS (Fig. 2 and 3). Dysregulation of iron homeostasis was indicated by the inhibition of Fur activity, resulting in enhanced levels of iron acquisition proteins (Table 1). The predicted increased influence of the general stress sigma factor RpoS was unexpected, since RpoS protein abundance decreased in the presence of vanillin (Fig. 2; see also Table 2). However, this anomaly could be resolved by analysis of the increased activity of DksA, which, along with ppGpp, acts to promote the association of alternative sigma factors with core RNA polymerase and hence the transcription of RpoS-dependent and, to a lesser extent, RpoN-dependent genes (35). Among the seven global transcription factors, only H-NS and Lrp were predicted to respond, with both exhibiting lower activity (Fig. 2; see also Table S4). Nevertheless, it was noted that 32 members of the 299-gene-strong Fnr regulon (36) were present in the significantly altered proteins listed in Tables 1 and 2. Most of these proteins decreased in abundance in the presence of vanillin, and all but 7 were shown to be coregulated by other TFs (Fig. 3). Hence, as implied by the TFInfer analysis, these additional regulators could be responsible for the observed changes. This is pertinent because all the cultures examined were grown under aerobic conditions and the level of Fnr activity would consequently be very low, but it is still possible that even this low level of activity could be further decreased by vanillin-promoted reactive oxygen species (ROS) production, linked to the response mediated by MarA, OxyR, and SoxS (see above), and that the altered abundance of the Fnr-activated proteins reported this change. While Fnr has a global influence over the metabolic mode seen in response to oxygen availability, several local regulators (CreB, ExuR, MalT, and UxuR) that are associated with carbon metabolism, as well amino acid-responsive regulators (ArgP, TrpR, and TyrR), were predicted to respond to the presence of vanillin, indicative of a perturbation of central metabolism (Fig. 2 and 3). Hence, these analyses revealed that vanillin invoked the following responses: (i) deployment of potential detoxification systems; (ii) changes in carbon metabolism; (iii) activation of an oxidative stress response; and (iv) perturbation of metal ion homeostasis. Therefore, these responses were further examined by additional physiological, biochemical, gene expression, and mutant studies.

### Detoxification of vanillin by reduction to vanillyl alcohol by YqhD and DkgA.

**FIG 4:**
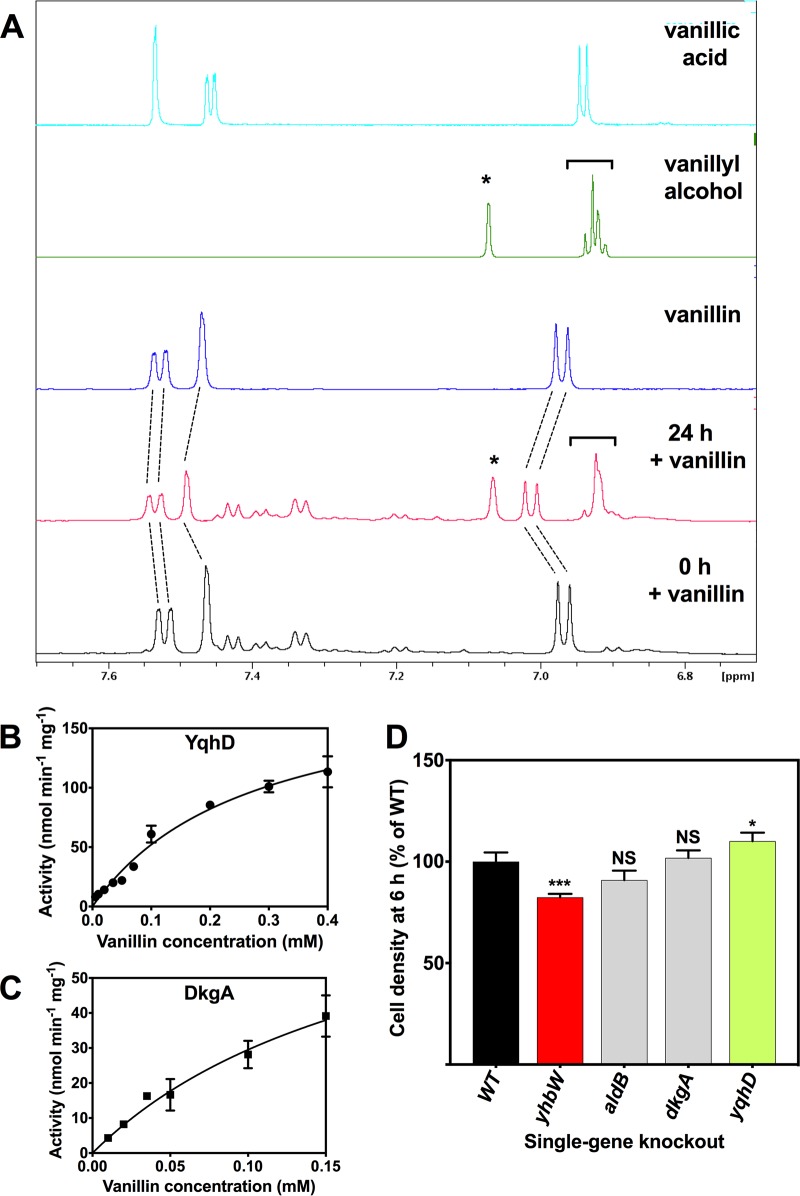
Vanillin detoxification by reduction to vanillyl alcohol. (A) ^1^H-NMR spectra of supernatants from cells incubated aerobically in LB for 0 h (black trace) or 24 h (red trace) with 10 mM vanillin. Reference spectra of vanillin (purple trace), vanillyl alcohol (green trace), or vanillic acid (light blue trace) are also shown. The dotted lines indicate the resonances of vanillin, which decreased in intensity after 24 h (the slight changes in chemical shift were probably due to pH changes in the culture during the incubation period). Resonances corresponding to the accumulation of vanillyl alcohol after 24 h are indicated (* and [). (B and C) Vanillin-dependent NADPH oxidation by purified recombinant YqhD (B) and DkgA (C). Data represent means and standard deviation of results from triplicate assays. Kinetic parameters are given in the text. (D) Comparison of levels of growth in LB plus 10 mM vanillin at 6 h after inoculation (OD_600_) of the BW25113 wild-type parent and single-gene-deletion strains from the Keio collection. Data represent means and standard deviation of results from triplicate cultures. A value of 100% corresponds to an OD_600_ of 0.49 ± 0.04. ***, *P* < 0.001; *, *P* < 0.05 (by one-way analysis of variance [ANOVA] compared to the WT). NS, not significant.

It has been suggested that inhibition of growth by aldehydes is caused by an NADPH drain, with aldehyde reductases consuming reductant at excessive rates (20–22). One consequence of NADPH depletion is reduced levels of cysteine and methionine production (22); however, this effect would be masked in our study by growth in rich media. Several enzymes previously shown to be involved in reducing certain aldehydes to alcohols were identified in the proteomics data set and were all more abundant during growth with vanillin (Table 1). In particular, the aldo-keto reductase DkgA (21) was ∼41-fold more abundant and the aldehyde reductase YqhD (20) was ∼35-fold more abundant. In the TFInfer analysis (Fig. 2), one of the largest changes in transcription factor activity was predicted for YqhC, an activator of the *yqhD-dkgA* operon (37). Interestingly, mutation of *yqhD* and *dkgA* has been shown to increase furfural tolerance in E. coli (21, 38), possibly by alleviating the NADPH drain. Ahr/YjgB and YahK (16, 39) were 8.5-fold and 3.6-fold more abundant, respectively (Fig. 1C; see also Table 1). The oxidoreductase YghA is annotated as uncharacterized but has been shown to have aldehyde reductase activity (16, 39) and was ∼4-fold more abundant. CurA, the NADPH-dependent curcumin reductase, also showed an ∼4-fold increase in abundance. Curcumin has a structure similar to that of vanillin and is also toxic to E. coli (40). In addition to these reductases, the aldehyde dehydrogenase AldB, which oxidizes aldehydes to carboxylic acids, was 7.8-fold more abundant during growth with vanillin. However, in agreement with previous work (16), ^1^H-NMR (^1^H nuclear magnetic resonance) spectroscopy showed that cells incubated aerobically with exogenous vanillin for 24 h accumulated vanillyl alcohol rather than vanillic acid (Fig. 4A), confirming that reduction is the predominant mode of vanillin metabolism in E. coli. Vanillyl alcohol is known to be less toxic to E. coli than vanillin (12).

As enzymes YqhD and DkgA were the most highly upregulated potential vanillin reductases during growth with vanillin (Table 1), we overproduced and purified recombinant YqhD and DkgA as C-terminal His-tagged proteins and demonstrated that both enzymes possessed NADPH-dependent vanillin reductase activity (Fig. 4B and C). With vanillin as the variable substrate, the overall kinetics were slightly better for YqhD (*K_m_*, 0.27 ± 0.04 mM; *V*_max_, 193 ± 15 nmol min^−1 ^mg^−1^; *k*_cat_ = 6.2 min^−1^; *k*_cat_/*K_m_* = 23 ± 1 min^−1 ^mM^−1^) than for DkgA (*K_m_*, 0.20 ± 0.08 mM; *V*_max_, 89 ± 25 nmol min^−1 ^mg^−1^; *k*_cat_ = 2.8 min^−1^; *k*_cat_/*K_m_* 14 ± 2 min^−1^mM^−1^). The level of activity with NADH was negligible for both proteins. Although YqhD and DkgA are clearly both highly upregulated by and can reduce vanillin, single-gene-knockout mutants in either *dkgA* or *yqhD* did not result in a decrease in vanillin tolerance compared to the wild-type (WT) strain (Fig. 4D). This is not surprising given the known large number of aldehyde reductases in E. coli (39) and is consistent with furfural toxicity studies reported previously (21, 38).

The uncharacterized protein YhbW exhibited the greatest increase in abundance (∼77-fold) of any protein during growth with vanillin. RT-PCR confirmed that *yhbW* expression was increased (by ∼13-fold) in cells grown in the presence of vanillin (Fig. S2). *yhbW* has been reported to be part of the SoxRS regulon, but upregulation was identified in a *soxS*-deficient strain at a level similar to that seen with the wild-type strain (Fig. S2). YhbW is homologous with luciferase-like alkanal monooxygenases (PFAM Pf00296) that use a reduced flavin mononucleotide (FMNH_2_) cofactor and molecular oxygen to oxidize their substrates. We therefore tested the hypothesis that this enzyme might oxidize vanillin to vanillic acid. YhbW was overproduced and purified as a recombinant C-terminal His-tagged protein, and assays were carried out under initially strictly anaerobic conditions with photoreduced FMN. However, no difference in activity was found after injection of either oxygen-saturated buffer or buffer plus vanillin. Nevertheless, a *yhbW* knockout mutant showed a small but significant decrease in cell yield during growth with vanillin compared to the WT (Fig. 4D), indicating that the enzyme does play some role in the response to vanillin.

10.1128/mSystems.00163-19.2 FIG S2 Vanillin-dependent regulation of *yhbW.* RT-PCR was used to assess expression of the *yhbW* gene in both wild-type and *ΔsoxS* mutant cells grown in the presence of 10 mM vanillin relative to untreated cells. Expression was normalized to that of the *rrsA* housekeeping gene. Data are plotted as means of results from three biological replicates (each consisting of three technical replicates). Error bars represent standard deviations. ***, <0.001 (as determined by Student’s *t* test). Download FIG S2, TIF file, 0.2 MB. Copyright © 2019 Pattrick et al. 2019 Pattrick et al. This content is distributed under the terms of the Creative Commons Attribution 4.0 International license.

### Pentose phosphate pathway and glyoxylate cycle enzymes are upregulated during growth with vanillin.

**FIG 5:**
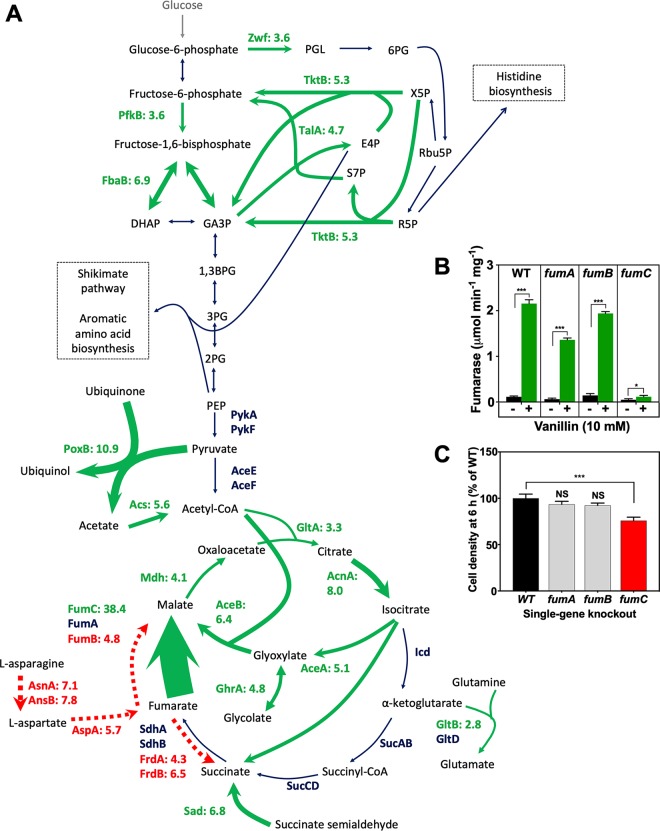
Changes in abundances of enzymes of central carbon metabolism during growth with vanillin and differential fumarase regulation. (A) Enzymes catalyzing the reactions shown in green were increased in abundance during growth with vanillin by the fold changes shown; enzymes catalyzing reactions shown in red were decreased by the fold changes shown. Enzymes catalyzing the reactions shown in dark blue showed no significant change in abundance. The thickness of the arrows is proportional to the fold change. PGL, 6-phosphogluconolactone; 6PG, 6-phosphogluconate; Rbu5P, ribulose-5-phosphate; R5P, ribose-5-phosphate; X5P, xylulose-5-phosphate; E4P, erythrose 4-phosphate; F6P, fructose 6-phosphate; S7P, sedoheptulose 7-phosphate; DHAP, dihydroxyacetone phosphate; GA3P, glyceraldehyde 3-phosphate; 1,3BPG, 1,3-bisphosphoglyceric acid; 3PG, 3-phosphoglyceric acid; 2PG, 2-phosphoglyceric acid; PEP, phosphoenolpyruvate. (B) Total fumarase activity of E. coli cell extracts from wild-type and fumarase-deficient cells grown in the absence or presence of 10 mM vanillin was measured spectrophotometrically at 240 nm. The data represent mean levels of activity of extracts from three independent cultures; error bars represent standard deviations. *, *P* = 0.01 to 0.05; **, *P* = 0.001 to 0.01; ***, *P* = <0.001 (by Student's *t* test). (C) Comparison of levels of growth in LB plus 10 mM vanillin at 6 h after inoculation (OD_600_) of BW25113 wild-type parent and single-gene-deletion strains from the Keio collection. Data represent means and standard deviations of results from triplicate cultures. A value of 100% corresponds to an OD_600_ of 0.49 ± 0.04. NS, not significant (compared to WT). ***, *P* < 0.001 (by one-way ANOVA compared to the WT).

Several of the enzymes described above that potentially catalyze the reduction of vanillin require NADPH; their upregulation and an increased flux of vanillin to vanillyl alcohol could lead to an undesirable increase in the NADP^+^/NADPH ratio, as proposed previously in reports of studies performed with other substrates (20–22). We found that enzymes of the pentose phosphate pathway (PPP), including glucose-6-phosphate dehydrogenase (Zwf), the transaldolase TalA, and transketolase TktB, were significantly increased in abundance during growth with vanillin (Table 1; see also Fig. 5A). The PPP is a major mechanism for regenerating NADPH (via Zwf), as well as for producing precursors for nucleotide and aromatic amino acid biosynthesis. z*wf* expression is controlled by the ferric uptake regulatory protein (Fur) and by MarA and SoxRS (36, 41), as shown in Fig. 2, providing a link with oxidative stress (see below). Interestingly, although TktA is the major transketolase in E. coli, its abundance was not significantly altered by vanillin treatment, whereas the abundance of the isoenzyme TktB, which is responsive to stress conditions via PhoB and RpoS regulation, was increased (Fig. 3). A similar situation occurs with the fructose-bisphosphate aldolase isoenzymes FbaA and FbaB, where FbaA is downregulated by osmotic stress (42). The proteomic data showed an ∼7-fold increase in FbaB abundance, with no change noted in FbaA levels during growth with vanillin. There appeared to be no changes in the levels of the Entner-Doudoroff pathway enzymes phosphogluconate dehydratase and 2-keto-3-deoxygluconate-6-phosphate aldolase. Evidence was also obtained indicating a shift from the complete oxidative citric acid cycle to use of the glyoxylate bypass. The glyoxylate bypass enzymes isocitrate lyase (AceA) and malate synthase (AceB) were upregulated 5-fold and 6.4-fold, respectively, during growth with vanillin (Table 1; see also Fig. 5A). Enzymes shared by both the citric acid cycle and the glyoxylate cycle—citrate synthase, aconitase and malate dehydrogenase—were also increased in abundance, while the citric acid cycle-specific enzymes isocitrate dehydrogenase (Icd), 2-oxoglutarate dehydrogenase (SucAB), and succinyl-CoA (succinyl-coenzyme A) synthetase (SucCD) were unchanged. The glyoxylate cycle requires two molecules of acetyl-CoA rather than the one required for the citric acid cycle. While AceE and AceF levels were unchanged, Acs increased in abundance (5.6-fold) during growth with vanillin. The only additional source of acetate in vanillin-treated cells thus appears to be from pyruvate via pyruvate oxidase (PoxB), reducing ubiquinone to ubiquinol in the process (Fig. 5A); PoxB was increased ∼11-fold in abundance during growth with vanillin. The enzyme GhrA, which catalyzes the reversible conversion of glycolate to glyoxylate, was 4.8-fold more abundant, while the enzymes GarR and GarD, which are involved in glyoxylate metabolism, were less abundant (Tables 1 and 2).

Several proteins whose involvement with respect to these central pathways was more peripheral also appeared to be involved in the vanillin response. The succinate semialdehyde dehydrogenase, Sad (YneI), was 6.8-fold more abundant, and knockout of the *sad* gene resulted in a 50% decrease in cell density in the presence of vanillin compared to the results seen with the wild type (see Fig. 9D), representing a greater effect than was shown by any other knockout mutant used in this study. Deletion of a neighboring gene that encodes an aminotransferase, *yneH*, also resulted in a significant decrease in cell yield in the presence of vanillin (see Fig. 9D). The corresponding two enzymes link glutamate metabolism pathways and the TCA cycle. AstC, which is involved in the degradation of arginine to glutamate and succinate, was also ∼4-fold more abundant in response to vanillin.

### Differential fumarase C upregulation avoids oxidative damage to FumA and FumB during growth with vanillin.

The class II fumarate hydratase FumC showed the third-highest fold increase in abundance (38.4-fold) of any protein in response to vanillin (Table 1; see also Fig. 1C and Fig. 5A), while the abundances of the class I fumarases FumA and FumB were unchanged and downregulated, respectively. FumC has been shown to act as a contingency enzyme for FumA and is upregulated primarily by the actions of Fur and SoxRS as a response to iron deficiency and superoxide accumulation, due to the fact that it is a stable tetramer that lacks the oxidant labile [4Fe-4S] cluster that FumA requires for catalysis (43, 44). To confirm and extend the protein abundance data for the three fumarases, fumarase activities in cell extracts derived from E. coli cultures grown with vanillin were compared to the levels seen with controls without vanillin (Fig. 5B). Strikingly, vanillin-grown wild-type, Δ*fumA*, and Δ*fumB* cells possessed similar, very high rates of activity; those rates were 50-fold to 100-fold higher than those seen in cells grown without vanillin. However, Δ*fumC* cells showed a very low level of activity which was similar to that seen with the wild type, irrespective of whether the cells were grown with vanillin or not, indicating that FumC was solely responsible for the increased vanillin-dependent fumarase activity. The importance of FumC was further highlighted by the fact that a Δ*fumC* mutant strain showed a significant increase in vanillin sensitivity (decreased final cell yield) compared to the wild type but that the Δ*fumA* and Δ*fumB* strains showed no significant change in growth (Fig. 5C). Taken together, the data from the proteomic, enzyme activity, and mutant growth assays show that vanillin-dependent upregulation of FumC is a key physiological response during growth in the presence of this compound. The use of FumC would result in avoidance of the enzyme damage to the FeS clusters of FumA or FumB that would be caused by reactive oxygen species (ROS), and it seems most likely that this striking form of differential regulation represents a response to increased oxidative stress during growth with vanillin. Note that the vanillin-dependent upregulation of the related dehydratase, aconitase A (AcnA), as opposed to AcnB (Table 1) also supports this conclusion, as the former is oxidative stress resistant whereas the latter is not (45). We further confirmed that vanillin causes oxidative stress (i) by measuring ROS production in vanillin-treated cells, (ii) by further analyzing the proteomic data, and (iii) by assessing changes in the vanillin tolerance of relevant mutants (see below).

### Vanillin causes accumulation of ROS and a global oxidative stress response.

**FIG 6:**
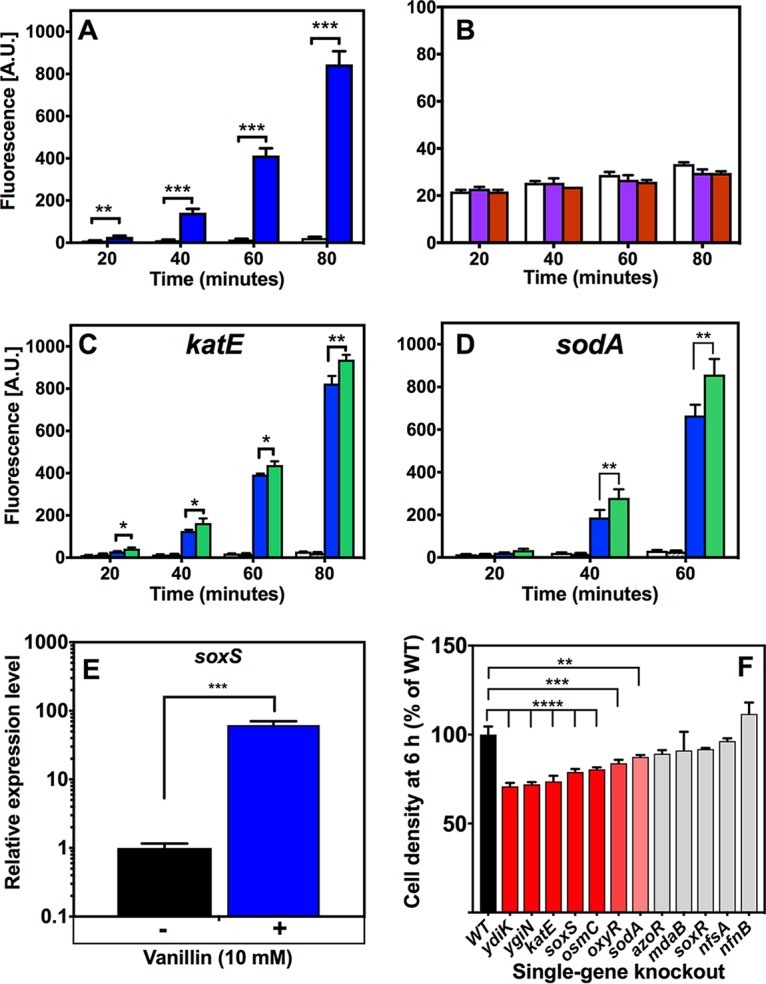
Vanillin treatment elicits reactive oxygen species (ROS) production and an oxidative stress response in E. coli. (A) Time-dependent increase in endogenous ROS levels in intact cells in response to 0.5 mM vanillin treatment (blue bars) detected by use of the ROS-activated fluorescent dye 2′,7′-dihydrodichlorofluorescein diacetate (H_2_DCFDA). The control without vanillin (white bars) showed no significant change. [A.U.], arbitrary units. (B) Same experiment as that described in the panel A legend but with 0.5 mM vanillic acid (purple bars) or vanillyl alcohol (red bars) compared to the control with no additions (white bars). Note the different fluorescence scale compared to panel A. (C and D) Experiments were performed in a manner similar to that described in the panel A legend with 0.5 mM vanillin, comparing the wild type (blue solid bars) with isogenic *katE* (C) or *sodA* (D) mutants from the Keio collection (green bars). (E) RT-PCR data showing changes in transcript levels of the oxidative stress response transcriptional activators *soxS* and *marA* in strains grown with and without 10 mM vanillin. Expression levels are expressed relative to the levels determined for untreated cells and normalized to the *rrsA* housekeeping gene. Data are plotted as means of results from three biological replicates (each consisting of three technical replicates) with standard deviations shown as error bars. (F) Comparison of levels of growth in LB plus 10 mM vanillin at 6 h after inoculation (OD_600_) of the BW25113 wild-type parent and the single-gene-deletion strains indicated (from the Keio collection). A value of 100% corresponds to an OD_600_ of 0.49 ± 0.04. In all panels, data are plotted as means of results from three biological replicates with standard deviations as error bars. *, *P* = 0.01 to 0.05; **, *P* = 0.001 to 0.01; ***, *P* < 0.001; ****, *P* < 0.0001 (Student's *t* test [A to E] or one-way ANOVA [F]).

Prediction of transcription factor activities in the absence and presence of vanillin clearly indicated increased activity of a subnetwork of local transcription factors (MarA, OxyR, and SoxS) involved in regulating the oxidative stress response (Fig. 2 and 3). A cell-permeant fluorescent indicator, 2',7'-dichlorodihydrofluorescein diacetate (H_2_DCFDA), was used to determine the level of production of endogenous ROS in response to vanillin and the related compounds vanillic acid and vanillyl alcohol. Treatment of cells with even low (0.5 mM) concentrations of vanillin caused a significant increase in the fluorescence of the probe relative to untreated cells (Fig. 6A). In contrast, vanillic acid or vanillyl alcohol treatment resulted in no apparent ROS production above the level seen with the untreated control (Fig. 6B). The use of such probes is convenient but has been criticized because they may not always indicate ROS production (46). However, additional evidence that vanillin does indeed cause ROS production is that the probe fluorescence was greater in E. coli strains with deletions in either of two key genes involved in oxidative stress defense, i.e., *sodA* and *katE* (Fig. 6C and D). Growth with vanillin also clearly increased the abundance of a large number of proteins involved in the oxidative stress response in E. coli (Table 1; see also Fig. 1C). Multiple proteins that are members of the SoxS regulon (41) were identified as being significantly upregulated as follows: YhbW (77.0-fold increase), FumC (38.4-fold), PoxB (10.9-fold), NfnB (14.8-fold), NfsA (12.8-fold), AcnA (7.9-fold), HemB (9.0-fold), MarR (9.4-fold), YgfZ (4.9-fold), and Zwf (3.6-fold). RT-PCR experiments showed that transcription of *soxS* itself was highly upregulated by vanillin (Fig. 6E), while a *soxS* deletion strain showed increased sensitivity to growth with 10 mM vanillin (Fig. 6F).

The response of E. coli to peroxide is largely mediated by the redox state of OxyR, with OxyR^ox^ activating transcription of several genes. These include the genes encoding alkyl hydroperoxide reductase subunits AhpC and AhpF, both of which were found to be more abundant during growth in the presence of vanillin (Table 1). The manganese-containing superoxide dismutase SodA is positively regulated by both SoxS and OxyR and showed an ∼5-fold increase in abundance, while the iron-containing superoxide dismutase SodB appeared to be unregulated by vanillin treatment. Mutants with deletions in *oxyR* and *sodA* were significantly impaired in growth with vanillin compared to the wild type (Fig. 6F). OxyR, along with Fur, induces expression of the *suf* operon that encodes proteins for iron sulfur cluster synthesis and repair (41). SufB, SufD, and SufS were all upregulated >5-fold by growth with vanillin (Fig. 1C; see also Table 1; see also Fig. 3).

Other proteome changes indicative of vanillin-dependent oxidative stress included proteins that are regulated indirectly or entirely independently of SoxRS and OxyR. The peroxiredoxin OsmC was upregulated 4.4-fold and the catalase KatE 4.8-fold. NfnB and MdaB, typically upregulated as a response to oxidative stress via MarR/MarA, showed 14.8-fold and 8.4-fold increases in abundance, respectively. Growth of mutants with deletions in *osmC* and *katE* was significantly impaired with vanillin compared to growth of the wild type, while no change was found with *nfnB* and *mdaB* mutants. (Fig. 6F). Several proteins key to glutathione (GSH) reduction and recycling—a process important in responses to oxidative, osmotic, and other stresses—also showed upregulation with vanillin (Fig. S3).

10.1128/mSystems.00163-19.3 FIG S3 Vanillin-dependent changes in abundance of glutathione cycling enzymes. The enzymes catalyzing the reactions represented in green showed an increase in abundance; the arrow thickness and numbers represent the fold increase exhibited during growth with vanillin. GSH, glutathione; GSSG, glutathione disulfide; GS-spermidine, glutathionylspermidine; GS-HQs, glutathionyl-hydroquinone; HQs, hydroquinone. Download FIG S3, TIF file, 0.4 MB. Copyright © 2019 Pattrick et al. 2019 Pattrick et al. This content is distributed under the terms of the Creative Commons Attribution 4.0 International license.

Among those proteins downregulated during growth with vanillin, there are many which again show that oxidative stress is an important feature of vanillin toxicity. Many proteins whose expression is controlled by the global oxygen-sensing anaerobic regulator Fnr, e.g., NirB, GlpA, GlpB, DmsA, HypB, NarH, NarG, AnsB, AspA, FumB, FrdA, and FrdB, showed a decrease in abundance (Table 2; see also Fig. 1C). A common feature in many of these and some other downregulated proteins, such as TdcG and SdaB (Table 2), is that they contain ROS-susceptible FeS clusters. In addition, two proteins that generate a highly ROS-sensitive and oxygen-sensitive glycyl radical required for their catalysis, GrcA (YfiD) and TdcE, were downregulated ∼5-fold and ∼61-fold, respectively (Table 2). One mechanism by which vanillin could cause ROS accumulation is through the impairment of ubiquinone biosynthesis. The chorismate pyruvate-lyase UbiC catalyzes the initial step of this pathway, and deletion of *ubiC* results in significant ROS production (47). UbiC is inhibited by vanillic acid and benzaldehyde (48) and thus likely also by vanillin. In addition, the major regulators SoxRS, MarA, and Rob bind and respond directly to structurally diverse aromatic compounds such as paraquat, salicylate, and bile salts, respectively (49). Vanillin may thus also interact with these regulators.

### Additional specific stress response pathways activated during growth with vanillin.

**FIG 7:**
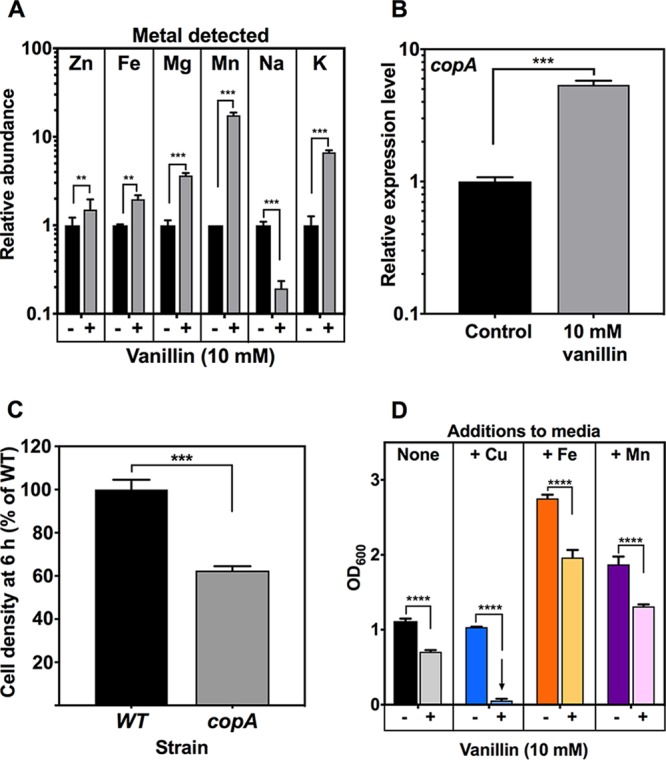
Vanillin-dependent changes in metal homeostasis. (A) Inductively coupled plasma mass spectrometry (ICP-MS) analysis of intracellular metal content of E. coli BW25113 in response to growth with 10 mM vanillin. (B) RT-PCR showing expression of *copA* in response to 10 mM vanillin relative to expression in untreated cells (control). Expression levels are normalized to the *rrsA* housekeeping gene. (C) Comparison of levels of growth in LB plus 10 mM vanillin at 6 h after inoculation (OD_600_) of BW25113 wild-type parent and isogenic *copA* deletion strain from the Keio collection. A value of 100% corresponds to an OD_600_ of 0.49 ± 0.04. (D) Cell density after 24 h growth of E. coli BW25113 in minimal medium alone (dark colored bars) or with 10 mM vanillin (light colored bars). Either no additional metal ions were added (None) or 5 μM copper II sulfate (+ Cu), 200 μM iron (II) sulfate (+ Fe), or 200 μM Mn (II) sulfate (+ Mn) was added. In each panel, the data shown represent means plus standard deviations of results from three independent cultures. *, *P* = 0.01 to 0.05; **, *P* = 0.001 to 0.01; ***, *P* < 0.001; ****, *P* < 0.0001 (Student's *t* test).

A number of other changes in protein abundance indicative of stress responses to growth with vanillin were observed. There was upregulation of multiple proteins that possess chaperone capabilities, indicating a response to protein damage. These included ClpB, CbpA, HslO, and HchA (Table 1). The proteolytic enzymes Dcp, PepB, and DegP were also upregulated, 3.1-fold, 5.1-fold, and 5.7-fold, respectively; DegP can also act as a chaperone. Upregulation of AcnA, HchA, HdhA, KatE, OsmC, OtsA, TalA, and TktB showed that they are indicators of an osmotic stress response. OtsA is a UDP-forming α,α-trehalose-phosphate synthase involved in the biosynthesis of trehalose, a low-molecular-weight (low-MW) compound that accumulates in response to high osmolarity (50). Moreover, TreB and TreC, two proteins vital to the degradative, catabolic utilization of trehalose as a carbon source, were ∼36-fold and ∼10-fold less abundant, respectively. Their expression is controlled by the TreR repressor (50). Other indicators of osmoprotectant accumulation include the increases in the abundances of glutamate synthase (GltB) and of the probable glutamate/gamma-aminobutyrate antiporter (GadC). The uncharacterized oxidoreductase YjhC was upregulated 23.8-fold; this enzyme has putative glucose-fructose oxidoreductase activity and is potentially involved in the production of sorbitol, another osmoprotective compatible solute (51). In support of these protein changes, inductively coupled plasma mass spectrometry (ICP-MS) analysis (Fig. 7A) showed an increase in intracellular K^+^ levels during exposure to vanillin, which is characteristic of an osmotic stress response.

### Metal homeostasis is perturbed in response to vanillin.

A vanillin-dependent increase in oxidative stress might be expected to be linked to a downregulation of the iron uptake machinery, given that careful iron management is essential due to intracellular Fenton chemistry facilitating production of damaging hydroxyl radicals and other ROS (52). However, the proteomic data showed that three enzymes of the pathway responsible for biosynthesis of the iron siderophore enterobactin, EntB, EntE, and EntF, were ∼37-fold, ∼8.5-fold, and ∼23-fold more abundant in cells grown with vanillin than in those grown without vanillin (Table 1). Other indicators of an absence of repressive Fur regulation were an increase in the abundance of the uncharacterized periplasmic protein YncE and examples of RyhB-mediated downregulation. RyhB is a noncoding RNA repressed by Fur that itself represses expression of iron-utilizing proteins, including FtnA, NirB, SodB, and FeS-containing enzymes of the TCA cycle (53). FtnA and NirB were found to be downregulated during growth with vanillin (Table 2). The upregulation of *suf* operon proteins, normally repressed by iron-bound Fur as well as being induced by OxyR, has been noted above. The perturbation of metal ion homeostasis in response to vanillin, mediated partly by changes in the activities of Fur and CueR as predicted in the TFInfer analysis (Fig. 2), is likely linked to attempts to repair oxidative damage to metallated proteins, including iron uptake and Suf-mediated iron-sulfur cluster repair.

In order to directly assess changes in metal homeostasis, changes in intracellular metal content in response to vanillin treatment were assessed by ICP-MS (Fig. 7A). We observed a significant increase in the intracellular levels of several transition metals detected in cells grown with vanillin. In particular, iron content increased modestly (∼2-fold), suggesting that the upregulation of the iron uptake machinery had a detectable effect. The largest vanillin-dependent change in metal content was a 17.5-fold increase in the level of manganese, a redox-active metal ion that acts as an antioxidant itself and is vital as a cofactor for the manganese-containing superoxide dismutase SodA. The proteomic data did not detect any proteins involved in manganese uptake, potentially due to the lower coverage of membrane proteins.

Although our ICP-MS analyses detected only very low copper levels, which were not accurately quantifiable, copper homeostasis is known to be intimately linked with oxidative stress management. It has been shown that Cu(I) ions destabilize FeS clusters, inhibit FeS assembly, and promote FeS cluster biogenesis, iron acquisition, and sulfur acquisition in bacteria (54, 55). This fits the apparent derepression of the Fur regulon shown with vanillin that occurs despite the accumulation of ROS. Copper efflux effects mediated via the Cus system and the P-type ATPase CopA are important means of coping with Cu(I)-mediated damage, especially to FeS clusters (54, 55). CopA is a central component of copper homeostasis and is regulated by CueR, activated by Cu(I) accumulation (56). We therefore used RT-PCR to measure the expression level of the *copA* gene and found it to be upregulated 5.4-fold in response to vanillin (Fig. 7B); the physiological effect of the presence of more CopA in the cell membrane would be increased transport of Cu(I) from the cytoplasm to the periplasm, where it can be oxidized to the less toxic Cu(II) form. A Δ*copA* strain showed significantly increased susceptibility to growth with vanillin treatment (Fig. 7C), suggesting an important role for CopA and copper homeostasis in vanillin toxicity. We further confirmed this by showing that the E. coli BW25113 wild-type strain was completely unable to grow in minimal medium in the presence of 10 mM vanillin in medium was supplemented with just 5 μM Cu(II), while supplementation with Fe(III) or Mn(II) caused growth stimulation in both the presence and absence of vanillin (Fig. 7D). Given that Cu(I) is the toxic species, we tested the hypothesis that vanillin itself could be acting to chemically reduce Cu(II) to Cu(I). We found that this was indeed the case (Fig. S4). This activity was also shown with the structurally related compounds vanillic acid, vanillyl alcohol, and ferulic acid but not with benzaldehyde, benzoate, 3-anisaldehyde (3-methoxy-benzaldehyde), and 4-hydroxy-benzaldehyde (Fig. S4). The data indicate that the presence of both the methoxy group on C4 and the hydroxyl group on C3 of the aromatic ring is required for this activity and that it is unrelated to the aldehyde moiety. Thus, we conclude that vanillin may directly disrupt copper homeostasis by increasing the ratio of Cu(I) to Cu(II), with the cells responding by increasing *copA* expression via CueR (Fig. 2).

10.1128/mSystems.00163-19.4 FIG S4 Copper reduction activities exhibited by vanillin and structurally related compounds. The Cu^+^-specific reagent BCS was used to assess the reductive effect of vanillin (A) and several aromatic compounds structurally related to vanillin (B to H). Activity was measured at a final concentration of 0.5 mM in all cases (black traces), except with vanillin (A) and vanillic acid (B), where activity was also measured at 1 mM (red traces). Download FIG S4, TIF file, 0.5 MB. Copyright © 2019 Pattrick et al. 2019 Pattrick et al. This content is distributed under the terms of the Creative Commons Attribution 4.0 International license.

### Vanillin specifically impairs maltose metabolism.

The proteomic data showed that two proteins involved in maltose uptake, i.e., the maltoporin protein (LamB) and the maltose periplasmic binding protein (MalE), were ∼17-fold and ∼15-fold less abundant, respectively, in E. coli cells grown with vanillin, and the activity of MalT (the activator of these genes) was predicted to decrease (Fig. 2 and 3). Downregulation of maltose transport in E. coli has previously been observed in response to toxic phenylpropanoids (57), which have a structure similar to that of vanillin. On the basis of those data, it was hypothesized that these transporters might be involved in vanillin’s movement into the bacteria; however, *lamB* and *malE* knockout mutants showed an increase and no change in vanillin sensitivity, respectively, rather than increased tolerance (Fig. S5A). Two other maltose-related proteins, MalQ (amlylomaltase) and MalP (maltose phosphotransferase component), were also less abundant in response to vanillin, indicating downregulation of the *malPQ* operon. In confirmation of these data, we found that E. coli growing in minimal medium with maltose as the sole carbon source was unable to grow in the presence of a concentration of vanillin that allowed growth on glucose (Fig. S5B). Vanillin thus renders E. coli specifically unable to utilize maltose as a carbon source.

10.1128/mSystems.00163-19.5 FIG S5 Vanillin impairs maltose utilization. (A) Comparison of levels of growth of the wild-type strain and the *lamB* and *malE* mutants in LB plus 10 mM vanillin at 6 h after inoculation (OD_600_). A value of 100% corresponds to an OD_600_ of 0.49 ± 0.04. (B) E. coli BW25113 cells were grown in M9 minimal media with either glucose or maltose as the sole carbon source and with or without 10 mM vanillin. Growth on maltose was reduced relative to growth on glucose was completely inhibited in the presence of vanillin. Data plotted represent means of results from three cultures with standard deviations shown as error bars, some of which are too small to be seen behind the symbols. Download FIG S5, TIF file, 0.5 MB. Copyright © 2019 Pattrick et al. 2019 Pattrick et al. This content is distributed under the terms of the Creative Commons Attribution 4.0 International license.

### Regulation and mutant phenotypes of relevant efflux systems.

**FIG 8:**
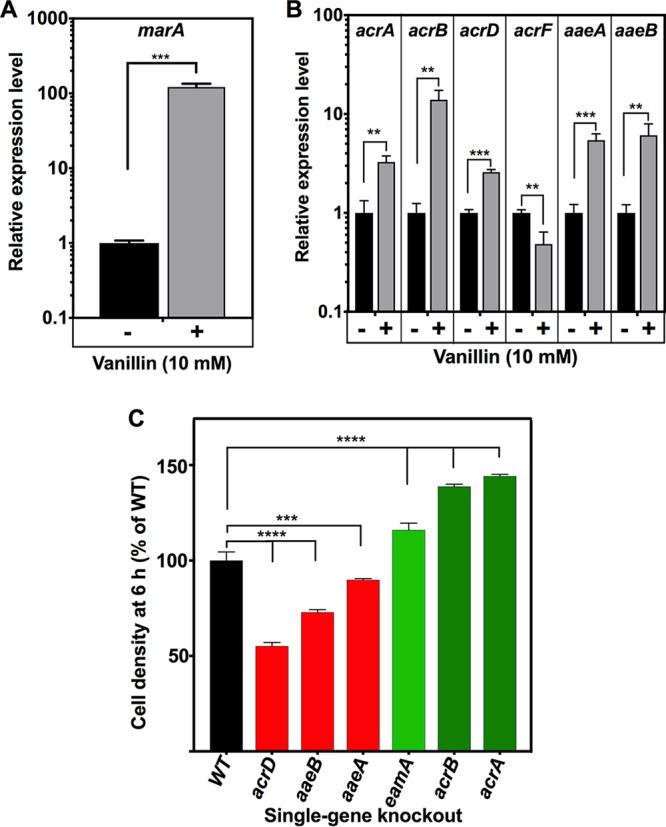
Gene expression and mutant phenotypes for genes encoding potential vanillin efflux systems. In (A and B) RT-PCR shows expression ratios for the genes shown in E. coli BW25113 cells treated with 10 mM vanillin relative to untreated cells, normalized to expression of *rrsA*. (C) Comparison of levels of growth in LB plus 10 mM vanillin at 6 h after inoculation (OD_600_) of BW25113 wild-type parent and single-gene-deletion strains from the Keio collection. A value of 100% corresponds to an OD_600_ of 0.49 ± 0.04. In each panel, the data represent means of results from three independent cultures with standard deviations shown as error bars. **, *P* = 0.001 to 0.01; ***, *P* < 0.001; ****, *P* < 0.0001 (Student's *t* test [A and B] or one-way ANOVA [C]).

One cellular strategy for alleviating vanillin toxicity could involve increased efflux. Due to low abundance, only a few of the membrane transport proteins that were directly identified from the proteomics data, for example, the multidrug efflux protein EmrA (part of the tripartite efflux system EmrAB-TolC) and the uncharacterized ABC transporter ATP-binding protein YadG, were significantly increased in abundance in response to vanillin. Expression of the major RND-type AcrAB-TolC multidrug efflux pump in E. coli is controlled by the MarR-MarA system. MarR can bind a range of compounds (including toxic aromatics), leading to activation of MarA, a transcriptional regulator, which (along with SoxS and Rob) modulates expression of many genes for efflux systems of broad specificity, including *acrAB* (58) and genes involved in oxidative stress and in organic solvent and heavy metal tolerance. MarR was upregulated ∼9-fold by vanillin (Table 1), and RT-PCR confirmed a large (∼100-fold) vanillin-dependent increase in transcription of the *marA* gene (Fig. 8A), consistent with the TFInfer modeling data that predicted a significant increase in MarA activity (Fig. 2). *acrA* and *acrB* were also upregulated (Fig. 8B), although knockout strains lacking either of the latter components of the AcrAB-TolC efflux system were actually less sensitive to (more tolerant of) vanillin than wild-type E. coli (Fig. 8B). Significantly, however, *acrD*, encoding an RND efflux pump protein that functions as an alternative to AcrB and controlled by the BaeSR two-component syetem (59), was also upregulated (Fig. 8B), and a Δ*acrD* strain did show significantly increased sensitivity to vanillin (Fig. 8C). The AaeAB aromatic acid efflux system (controlled by AaeR) expels aromatic carboxylates; *aaeA* and *aaeB* genes were vanillin upregulated (Fig. 8B), and both the Δ*aaeA* and Δ*aaeB* strains showed an increased sensitivity to vanillin compared to the wild type (Fig. 8C). Taken together, these data suggest that AcrD and AaeAB are most likely components of vanillin efflux systems, while active AcrAB seems to be associated with vanillin sensitivity. The increase in vanillin tolerance in *acrA* and *acrB* mutants is similar to that seen for some other biotechnologically relevant products such as isoprenol and isobutanol (60–62). It is most likely caused by the known upregulation of genes for other efflux proteins such as *acrD* in such strains (42, 63). AcrD has been implicated in both drug and metal resistance (59). AaeAB in E. coli acts as a system for relieving the toxicity that results from the accumulation of aromatic acids from metabolism such as *p-*hydroxybenzoate; its expression is induced by the presence of some other aromatics such as salicylate (64). No efflux of aromatic aldehydes via this system has been reported, but this is clearly an excellent vanillin efflux candidate.

### Generation and analysis of vanillin-tolerant strains by adaptive laboratory evolution (ALE).

**FIG 9:**
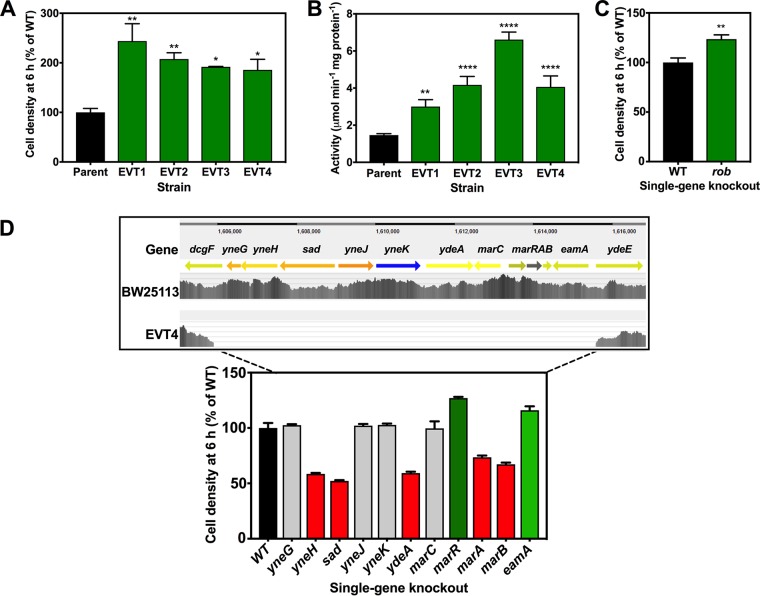
Phenotypic analysis of EVT strains. (A) Comparison of levels of growth in LB plus 10 mM vanillin at 6 h after inoculation (OD_600_) of BW25113 wild-type parent and EVT strains 1 to 4. A value of 100% corresponds to an OD_600_ of 0.49 ± 0.04. (B) Specific activity of citrate synthase (GltA) in wild-type and EVT strain cell extracts (cells were grown in LB without vanillin). (C) Comparison of levels of growth in LB plus 10 mM vanillin at 6 h after inoculation (OD 600 nm) of BW25113 wild-type parent and a single gene deletion mutant in the *rob* gene, derived from the Keio collection. A value of 100% corresponds to an OD_600_ of 0.49 ± 0.04. In panels A to C, the data represent means of results from three independent cultures or assays, with standard deviations shown as error bars. *, *P* = 0.01 to 0.05; **, *P* = 0.001 to 0.01; ****, *P* < 0.0001 (one-way ANOVA [A and B] or Student's *t* test [C]). (D) The ∼10-kb chromosome deletion in EVT4 and the effect of individual single-gene knockouts in this region on vanillin tolerance. (Upper panel) The 9,699-bp region deleted in EVT4, encompassing genes *yneG* to *eamA* with partial deletion of *dcgF*, as shown by the sequence read density (gray vertical bars). (Lower panel) Comparison of levels of growth in LB plus 10 mM vanillin at 6 h after inoculation (OD_600_) of BW25113 wild-type parent and single-gene-deletion strains from the Keio collection. A value of 100% corresponds to an OD_600_ of 0.49 ± 0.04. Red bars indicate significantly decreased growth (*P* < 0.05 by one-way ANOVA), and green bars significantly increased growth (*P* < 0.05 by one-way ANOVA) compared to the wild type. Gray bars indicate no significant change.

Four strains were isolated following repeated, serial subculturing of E. coli BW25113 in gradually increasing concentrations of vanillin in rich media (see Materials and Methods). They were named evolved vanillin tolerance (EVT) strain 1 (EVT1) to EVT4. Panel A of Fig. 9 shows that these strains grew significantly better in the presence of vanillin than the parent strain, BW25113. The genomes of these strains were sequenced and compared to that of the parent strain, which had been subcultured identically but in the absence of vanillin. No single nucleotide polymorphisms (SNPs) or other genome changes were found in the latter strain after the same number of subcultures. However, several SNPs and deletions in coding regions were indentified in EVT1 to EVT4 (summarized in Table 3).

**TABLE 3:** Genomic changes identified in EVT E. coli strains^*a*^

Strain	Position (bp)	Change	Gene(s)	Description
EVT1	749566	R119L (CGT→CTT)	*gltA*	Citrate synthase
	4625115	Δ1 bp, coding (12/870 nt)	*rob*	Right origin-binding protein

EVT2	748772	A384T (GCA→ACA)	*gltA*	Citrate synthase
	1612766	Δ11 bp, coding (389–399/666 nt)	*marC*	Inner membrane protein

EVT3	749516	G136S (GGT→AGT)	*gltA*	Citrate synthase
	4624518	Q203stop (CAG→TAG)	*rob*	Right origin-binding protein
	3169702	L162del (CTGCAT→CAT)	*cpdA*	cAMP phophodiesterase

EVT4	749443	A160V (GCC→GTC)	*gltA*	Citrate synthase
	1605879	Δ9,699 bp	12 genes	*dgcF* to *eamA*

a EVT, evolved vanillin tolerant; nt, nucleotide.

Most strikingly, all four EVT strains had distinct nonsynonymous SNPs in the citrate synthase gene, *gltA*. GltA in E. coli forms a hexamer, and the R119L and G136S mutations in EVT1 and EVT3, respectively, occur at the interface between GltA monomers (Fig. S6A, B, E, and F). The A348T mutation in EVT2 occurs in the region of the acetyl-CoA binding site and is next to a phenylalanine residue (F383) that forms part of this site (Fig. S6C and D). Finally, the A160V mutation in EVT4 occurs next to an isoleucine residue (I159) that is part of the allosteric NADH binding site (Fig. S6G and H). We initially assumed that the *gltA* mutations in EVT1 to EVT4 might negatively affect GltA activity. However, enzyme assays carried out with cell extracts of strains EVT1 to EVT4 grown without vanillin showed that each strain had increased specific activity compared to the wild-type strain, which had been subcultured in parallel in the absence of vanillin (Fig. 9B). In order to determine if the mutations were directly responsible for the increased GltA activity and did not signify, e.g., changes in protein abundance, we cloned and overexpressed wild-type and mutant *gltA* genes in pET-21a/E. coli BL21(DE3) (see Materials and Methods). We then purified the recombinant His-tagged wild-type GltA and R119L, A348T, G136S, and A160V variant enzymes and carried out a kinetic analysis with oxaloacetate as the variable substrate at a fixed acetyl-CoA concentration (Table 4). The results showed that the oxaloacetate *K_m_* values were ∼3-fold to ∼4-fold lower with each variant enzyme than with the wild-type enzyme and that the *k*_cat_ values were more similar, giving ∼2-fold to ∼4-fold higher *k*_cat_/*K_m_* values for the variants than for the wild type.

**TABLE 4:** Kinetic parameters determined for purified recombinant wild-type and variant citrate synthases with mutations matching those in strains EVT1 to EVT4^*a*^

Citrate synthase	*K_m_* (μM)	*V*_max_ (μmol min^−1^ mg protein^−1^)	*K*_cat_ (min^−1^)	*K*_cat_/*K*_m_ (min^−1^ mM^−1^)
WT	154 ± 25	4.8 ± 0.3	232 ± 14	1,506 ± 88
R119L (EVT1)	45 ± 12	5.6 ± 0.4	268 ± 18	5,955 ± 613
A384T (EVT2)	62 ± 6	4.0 ± 0.1	192 ± 7	3,096 ± 119
G136S (EVT3)	37 ± 4	4.5 ± 0.1	214 ± 7	5,783 ± 90
A160V (EVT4)	42 ± 7	4.2 ± 0.2	202 ± 10	4,809 ± 181

a The data shown are means and standard deviations of at least three independent rate measurements over a range of oxaloacetate concentrations at a fixed concentration of acetyl-CoA, using the DTNB assay for CoA formation (see Materials and Methods).

10.1128/mSystems.00163-19.6 FIG S6 Locations of mutations in GltA in EVT strains. (A and B) Change from R119 (red) to L119 (gray) in EVT1 GltA. GltA forms a hexamer, and the panels show the two layers of dimer pairs (light green-dark green, light blue-dark blue, pink-brown) and the location of residue 119 at dimer interfaces. (C and D) Change from A384 (red) to T384 (gray) in the vicinity of the oxaloacetate binding pocket (oxaloacetate modeled in purple) in EVT2 GltA. (E and F) Change from G136 (red) to S136 (gray) at the interface between the two monomers (shown in dark blue and light blue) in EVT3 GltA. (G and H) Change from A160 (red) to V160 (gray) in a helix near the NADH allosteric binding site (NADH modeled in red) in EVT4 GltA. Images were created in PyMOL using PDB entries 1OWB (panels A, B, E, F, G, and H) and 4JAG (panels C and D). The data shown represent means and standard deviations of at least three independent rate measurements over a range of oxaloacetate concentrations at a fixed concentration of acetyl-CoA, using the DTNB assay for CoA formation (see Materials and Methods). Download FIG S6, TIF file, 2.6 MB. Copyright © 2019 Pattrick et al. 2019 Pattrick et al. This content is distributed under the terms of the Creative Commons Attribution 4.0 International license.

Crystal structures of the variant enzymes are required to provide a detailed explanation of how such changes affect activity, but it is interesting that some known mutations seen in the vicinity of some of those found here have the opposite effect, for example, an F383A mutation reduced substrate turnover (65) and an A161V mutant had an increased *K_m_* level for oxaloacetate and decreased overall activity (66, 67). Given that all the EVT strains had GltA variants with improved activity and that there was an ∼3-fold increase in GltA abundance in unevolved wild-type cells during growth with vanillin (Table 1), we conclude that increased GltA activity is an important factor in vanillin tolerance. It is significant that, in unrelated long-term E. coli evolution experiments where higher growth rates were selected for under specific conditions, mutations in *gltA* arose and initially increased enzyme activity; these may act as “enabling” mutations in concert with other mutations by allowing better fitness as a consequence of increased citric acid cycle or glyoxylate cycle flux (66). This may explain the presence of the additional mutations in each EVT strain in addition to the *gltA* mutations.

Interestingly, EVT1 and EVT3 had mutations in the *rob* gene, which encodes an AraC/XylS-family transcriptional regulator. Rob recognizes a DNA binding site similar to that of SoxS and MarA, and their regulons overlap (49). The frameshift caused by the single nucleotide deletion in the *rob* gene in EVT1 results in a premature stop codon and a severely truncated product of just nine amino acids. The SNP in EVT3 also causes a premature stop codon, preventing translation of the 85 C-terminal amino acids. Both these mutations completely block or severely impair Rob-dependent regulation. We found that a *Δrob* knockout mutant showed increased vanillin tolerance (Fig. 9C), suggesting that the *rob* mutations in EVT1 and EVT3 do indeed contribute to their vanillin tolerance phenotype. EVT3 also had a 3-bp deletion in *cpdA*, which encodes a 3′,5′-cyclic AMP phosphodiesterase. This resulted in an in-frame deletion of a leucine residue (Leu162) that is semiconserved in bacterial cAMP phosphodiesterases.

An 11-bp deletion was identified in the *marC* gene in EVT2. The function of MarC is unknown, but it has been suggested as a candidate alcohol channel or pore protein (68, 69), and *marC* expression is controlled by the MarA regulator. Interestingly, the Keio collection *marC* deletion mutant did not show in any change in vanillin growth sensitivity (Fig. 9D, lower panel). Therefore, we conclude that the *gltA* mutation in EVT2 is responsible for the increased tolerance of vanillin in this strain.

Finally, EVT4 had an ∼10-kb deletion that removed 12 genes (11 were entirely deleted, and *dgcF* was partly deleted), including *marC* and the entire *marRAB* operon (Fig. 9D, upper panel). We compared the effects on growth with vanillin of single deletions in each of these genes with the WT (Fig. 9D, lower panel) and EVT4 (Fig. 9A). Deletions in *sad*, *yneH*, *ydeA*, *marA*, and *marB* caused slower growth with vanillin than was seen with the WT. Increased growth with vanillin was seen with single-gene knockouts of *marR* and *eamA* (Fig. 9D, lower panel). The effect of deleting *marR* reinforces the idea of a role of *marRAB*-mediated regulation in vanillin stress. The gene *eamA* encodes a putative cysteine (and cysteine pathway metabolite) efflux pump, the deletion of which could increase the size of the intracellular cysteine pool. One major outcome of normal regulation via the MarRA regulon is increased production of the AcrAB efflux pump, with this upregulation previously having been shown to be Rob mediated (70). Given that the *ΔacrA* and *ΔacrB* strains showed increased vanillin tolerance, a rationale for mutations in *rob* and *marR* in the EVT strains is prevention of AcrAB expression, which contributes to vanillin tolerance. Interestingly, two of six evolved isopropanol-tolerant strains of E. coli also showed large deletions (consisting of 25 and 34 genes) in the region of the genome covering the ∼10,000 bp deleted in EVT4 (71).

### Postscript: exogenous vanillin addition versus endogenous production.

In this study, we focused on the response of E. coli to exogenous vanillin addition, in order to identify the major responses to and toxic effects of this molecule. This approach is easily controllable and experimentally convenient. However, it would clearly be desirable to study toxicity effects in a strain producing vanillin endogenously. We attempted to construct such a strain, using the system described by Yoon et al. (11), who reported high levels (∼1 to ∼2 g/liter) of vanillin production from ferulate via ferulate:CoA ligase (Fcs) and enoyl CoA hydratase/lyase (Ech) derived from *Amycolatopsis*. Here, the corresponding *fcs* and *ech* genes were synthesized commercially and subjected to codon optimization for E. coli. After cloning into pTRC (*trc* promoter), pSTV (*tac* promoter), or pBAD (arabinose inducible promoter) vector and expression in DH5α, BL21(DE3), and BW25113 strains, production of vanillin was tested as described previously by Yoon et al. (11) in yeast extract-tryptone (YT) medium plus ferulate at 2 g/liter, with the medium analyzed by high-performance liquid chromatography (HPLC). However, no vanillin production or ferulate consumption was detected, despite each strain producing the Fcs and Ech proteins as judged by SDS-PAGE gel profiles. *fcs* and *ech* genes comprising a second set were cloned from Pseudomonas putida S12 into the pBAD vector and used to transform the same set of E. coli strains. These genes are homologous to the *fcs*/*ech* genes used previously by Barghini et al. (12) in another vanillin production study. While the two proteins could again be detected by SDS-PAGE, no vanillin was produced after arabinose induction when the cells were grown on YT-ferulate medium. We cannot explain why these approaches failed but are reporting these negative results to inform other researchers attempting to construct vanillin-producing E. coli strains.

### Conclusions.

In this report, we present novel insights into vanillin toxicity in E. coli. From the proteomic data and supporting physiological experiments, a perturbation of key central metabolic pathways and metal homeostasis was revealed and several vanillin detoxification and potential efflux systems were identified. We also found evidence of an oxidative stress response during growth with vanillin, despite the reported antioxidant characteristics of this molecule (72). The increased expression of *copA* and the copper growth sensitivity data suggest that the presence of vanillin resulted in Cu(I)-mediated toxicity, most likely as a result of the ability of vanillin to reduce Cu(II). Modeling of TF activity based on the protein abundance data revealed the key regulatory systems involved in the response to vanillin. It seems highly likely that vanillin can interact with and activate one or more TFs, leading to many of the changes in protein abundance that we observed (see Fig. 3). The changes in the genomes of the evolved vanillin-tolerant E. coli strains gave additional novel insights into the long-term adaptive responses needed to allow better growth with this substrate. A key feature in each strain was the presence of mutations leading to increased citrate synthase activity. The detection of mutations in the *rob* gene in EVT1 and EVT3 and the of deletion of the *marRAB* operon in EVT4, plus the increased vanillin tolerance shown by the *Δrob* and *ΔmarR* Keio strains, underlines the importance of the Rob and MarRA regulators in vanillin tolerance.

It should be noted again that this study focused solely on the effect of exogenous addition of vanillin and that the results may be different from those responses seen when vanillin is produced endogenously. Nevertheless, our results suggest several possibilities for engineering solutions to improve vanillin tolerance where E. coli is being used as a cell factory for production (11–13). The significant increase in intracellular ROS implies that overexpression of oxidative stress defense enzymes might prove effective, while growth at low copper levels and overexpression of CopA could potentially alleviate vanillin-mediated Cu(I) toxicity. Transcription factor engineering based on the networks identified here can be applied, as has been done to improve solvent tolerance in E. coli (73). Engineering effective endogenous efflux systems for vanillin in E. coli would result in an increased tolerance of synthesized vanillin while also potentially improving product recovery. The proteomic data and vanillin sensitivity of mutants suggest that the AaeAB efflux pump and AcrD are good starting points. Vanillin is just one of many lignin-derived phenolic compounds of biotechnological interest where toxicity might limit yields. Our approaches can be applied more generally to such compounds to investigate toxicity mechanisms, with the knowledge gained aimed at generating more extensively product-tolerant strains.

## MATERIALS AND METHODS

### Bacterial strains and growth conditions.

E. coli BW25113, a derivative of F^−^ λ^−^ E. coli K-12 strain BD792, was used as the standard wild-type strain. Single-gene-knockout strains were obtained from the Keio collection (26) maintained at The University of Sheffield, United Kingdom. E. coli cultures were typically grown aerobically in lysogeny broth (LB) at 37°C in 50-ml cultures in 250-ml conical flasks shaken at 200 to 250 rpm. For growth in minimal media, E. coli was grown in M9 medium supplemented with 1.8 μM thiamine and a 0.2% (wt/vol) carbon source. Antibiotics were used at the following concentrations: carbenicillin, 100 μg/ml; kanamycin, 50 μg/ml. Vanillin was dissolved as a concentrated stock solution in dimethyl sulfoxide (DMSO), and the same volume of DMSO was used in control cultures. Vanillin sensitivity of wild-type and mutant strains was assessed by comparing levels of growth in LB plus 10 mM vanillin as the optical density at 600 nm (OD_600_) after 6 h postinoculation in 50-ml shaken cultures in 250-ml conical flasks. For testing vanillin production in engineered strains, E. coli strains were grown in 2× yeast extract-tryptone (2YT) medium supplemented with 2 g/liter ferulate (11) and the appropriate antibiotic for plasmid maintenance. Overnight cultures of the strain were diluted in fresh 2YT-plus-ferulate medium (50 ml in 250-ml conical flask) to an OD_600_ of 0.1. To induce protein production, the medium contained either 1 mM IPTG (isopropyl β-d-1-thiogalactopyranoside) (for pTRC and pSTV plasmids) or 0.2% wt/vol l*-*arabinose (pBAD plasmids). Cultures were incubated at 37°C with shaking at 200 to 250 rpm. Samples were taken for HPLC analysis at 0 h and after 24 h of incubation.

### HPLC analysis.

Culture samples (1 ml) were prepared for HPLC analysis by centrifugation (12,000 × *g*, 5 min) and filtration (0.2-μm-pore-size nitrocellulose filter) of the supernatants. Ferulate and vanillin were quantified using a Waters e2695 HPLC system equipped with a photodiode array (PDA) detector set at a single wavelength of 260 nm. Analytes were resolved on a Waters Xbridge C_18_ column (3.5-μm pore size, 4.6 mm by 3.5 mm) at 25°C. Mobile phase A was 20 mM sodium acetate (pH 6), and mobile phase B was methanol (100%). The mobile phase regime started at 100% A. The ratio of phase A to phase B was increased linearly to 50:50 at 7 min and to 10:90 at 8 min; this was maintained for 30 s until returning to 100% phase A at 9 min. Authentic standards of vanillin and ferulate (Sigma-Aldrich) had retention times of 6.1 min and 3.8 min, respectively.

### Peptide sample preparation for mass spectrometry.

E. coli BW25113 cultures were grown overnight in 50 ml LB in 250-ml conical flasks at 37°C and were used to inoculate 1 liter LB in 2-liter flasks with a starting OD_600_ of 0.1. Cultures were grown either in the absence of vanillin or in the presence of 10 mM vanillin to an OD_600_ of 0.5 and harvested by centrifugation at 10,000 × *g* for 10 min at 4°C. Cell pellets were washed twice with 20 mM sodium phosphate buffer (pH 7.4) and stored at −80°C until use. A cell extract was prepared by sonication, and the protein concentration was measured by Bradford assay. A total of 50 μg of protein was added to each well of a 12.5% (wt/vol) acrylamide SDS-PAGE gel and run at 160 V for 15 min. The gel was stained with colloidal Coomassie (Invitrogen) for 1 h and destained with 25% (vol/vol) methanol for 1 h, and each lane was divided into 6 gel fractions and cut into 2-mm^3^ sections. Gel slices were reduced with 10 mM dithiothreitol (DTT) at 56°C for 1 h and alkylated with 55 mM iodoacetamide in the dark, at room temperature, for 30 min. Proteins were digested with 120 ng Pierce trypsin protease for 1 h at 37°C with gentle agitation followed by overnight incubation at 25°C. Peptides were extracted from gel fractions by treatment with pure acetonitrile followed by treatment with 0.5% (vol/vol) formic acid, after which the supernatant was transferred to a peptide collection tube. This process was repeated three times before a final treatment with pure acetonitrile was performed. Finally, the samples were dried using a SpeedVac. For mass spectrometric analysis, samples were resuspended in 0.5% (vol/vol) formic acid.

### Mass spectrometry analysis.

Extracted peptides were analyzed by nano-liquid chromatography tandem mass spectrometry (nano-LC-MS/MS) on an Orbitrap Elite (Thermo Fisher) hybrid mass spectrometer equipped with a nanospray source and coupled with an Ultimate RSLCnano LC system (Dionex). The system was controlled by the use of Xcalibur 2.1 (Thermo Fisher) and DCMSLink 2.08 (Dionex). Peptides were desalted on-line using a micro-Precolumn cartridge (C_18_ Pepmap 100; LC Packings) and then separated using a 60-min reversed-phase gradient (4% to 32% [vol/vol] acetonitrile–0.1% [vol/vol] formic acid) on an Easy-Spray column 15 cm by 50-μm inner diameter [ID], PepMap C_18_, 2-μm-diameter particles, 100-Å pore size; Thermo). The Orbitrap Elite was operated with a cycle of one MS (in the Orbitrap) acquired at a resolution of 60,000 at *m*/*z* 400, with the top 20 most abundant multiply charged ions (2^+^ and higher) in a given chromatographic window subjected to MS/MS fragmentation in the linear ion trap. A Fourier transform mass spectrometry (FTMS) target value of 1E−6 and an ion trap MSn target value of 1E−4 were used with the lock mass (445.120025) enabled. A maximum FTMS scan accumulation time of 500 ms and a maximum ion trap MSn scan accumulation time of 100 ms were used. Dynamic exclusion was enabled with a repeat duration of 45 s with an exclusion list value of 500 and exclusion duration of 30 s.

### Proteomics data analysis and modeling.

MS data were analyzed using MaxQuant version 1.5.2.8 (74). Data were searched against an E. coli UniProt sequence database using the following search parameters: 2 missed cleavages (maximum) for trypsin, 7 ppm for MS mass tolerance, and 0.5 Da for MS/MS mass tolerance, with acetyl (protein N terminus) and oxidation (M) set as variable modifications and carbamidomethylation (C) set as a fixed modification. A protein false-discovery rate (FDR) of 0.01 and a peptide FDR of 0.01 were used for identification-level cutoffs. Label-free quantification was performed using MaxQuant calculated protein intensities with matching between runs (with a 2-min retention time window) enabled (75). Data were filtered such that at least 3 valid LFQ intensity values were present in either group and were normalized by subtraction of the median value corresponding to each group. Missing values were imputed using Perseus (1.4.1.3), and two-sample *t* tests were performed with a permutation-based FDR calculation in Perseus (31). Fold changes (vanillin treated/control) were calculated from averaged normalized intensities after imputation. Inference of transcription factor (TF) activities from the proteomic changes using TFInfer 1.0 (32) was achieved by combining a connectivity matrix consisting of 2,137 genes and 194 TFs with a file containing fold changes in LFQ intensity values for 1,052 proteins detected under both sets of conditions.

### RNA extraction and qRT-PCR.

E. coli cultures were grown to an OD_600_ of 0.5 and centrifuged at 10,000 × *g*, and the pellets were resuspended in TE buffer (10 mM Tris-HCl, 1 mM EDTA, pH 8) with 1 mg ml^−1^ lysozyme (Sigma-Aldrich) added. Following incubation for 5 min, RNA extraction was performed using an SV total RNA isolation system kit (Promega). Purified RNA underwent a second DNase treatment step using a Turbo DNA-free kit (Ambion) and was stored at −80°C until required. Reverse transcription-quantitative PCR (qRT-PCR) was performed using a SensiFAST SYBR Lo-ROX one-step kit (Bioline) in 96-well optical reaction plates, with each reaction performed in triplicate. Primers for genes of interest (listed in Table S1 in the supplemental material; XXX_RTPCR_F and XXX_RTPCR_R, where XXX is the gene name) were designed for ∼200-bp amplicons, with a melting temperature (*T_m_*) of ∼60°C. Each reaction consisted of 10 μl of 2× Sensimix SYBR-Lo-ROX, 0.2 μl reverse transcriptase, 0.4 μl RNase inhibitor, 0.25 μM primers, and 20 ng template RNA, made up to reach a total volume of 20 μl with nuclease-free double-distilled water (dH_2_O). A standard curve for each pair of primers was produced using E. coli genomic DNA, and gene expression was calculated relative to expression of the reference gene, *rrsA* (76). PCR amplification was performed using a Stratagene MX3005p thermal cycler (Agilent). The cycling protocol was as follows: 10 min at 45°C; 2 min at 95°C; and 40 cycles of 20 s at 95°C, 30 s at 55°C, and 20 s at 72°C. Threshold cycle (*C_T_*) values were determined by the use of MxPRO quantitative PCR (qPCR) software (Agilent). and relative gene expression levels were calculated using the comparative method described in User bulletin 2: ABI Prism 7700 sequence detection system (https://www.core-facility.uni-freiburg.de/qpcrddpcr/lc480obj/ub2).

10.1128/mSystems.00163-19.7 TABLE S1 Primers used in this study. Download Table S1, PDF file, 0.06 MB. Copyright © 2019 Pattrick et al. 2019 Pattrick et al. This content is distributed under the terms of the Creative Commons Attribution 4.0 International license.

10.1128/mSystems.00163-19.8 TABLE S2 Proteins identified from label-free shotgun proteomic analysis by LC-MS/MS. The protein group file generated by analysis of the raw MS files using MaxQuant is shown. It includes the complete set of 1,885 proteins identified at a false-discovery rate (FDR) of 1% at the peptide and protein levels. Download Table S2, XLSX file, 2.3 MB. Copyright © 2019 Pattrick et al. 2019 Pattrick et al. This content is distributed under the terms of the Creative Commons Attribution 4.0 International license.

10.1128/mSystems.00163-19.9 TABLE S3 Statistical analysis of the label-free quantification (LFQ) data using Perseus. The list of 850 proteins that were reproducibly quantified is shown with the corresponding normalized LFQ intensities. Imputed protein intensity values are highlighted in yellow, and a plus sign (+) indicates that the abundances of proteins in the control and vanillin-grown samples were significantly different as assessed by *t* testing with a permutation FDR of 0.05. Download Table S3, XLSX file, 0.3 MB. Copyright © 2019 Pattrick et al. 2019 Pattrick et al. This content is distributed under the terms of the Creative Commons Attribution 4.0 International license.

10.1128/mSystems.00163-19.10 TABLE S4 Raw output of TFInfer. A connectivity matrix derived from the known regulons of the transcription factors in E. coli was compiled from data reported previously by Gama-Castro et al. (36). The fold changes in the abundances of the proteins listed in Table 1 and Table 2 were used as the input for analysis by TFinfer (Asif et al. [32]) to infer the activity of these transcription factors. Download Table S4, XLSX file, 0.02 MB. Copyright © 2019 Pattrick et al. 2019 Pattrick et al. This content is distributed under the terms of the Creative Commons Attribution 4.0 International license.

### ^1^H-NMR analysis of vanillin metabolism.

Cultures of E. coli BW25113 were grown aerobically in LB with 10 mM vanillin. Samples were taken immediately after inoculation (0 h) and at 24 h. Cells were removed by centrifugation (13,800 × *g* for 5 min) and the supernatants frozen at −20°C. After thawing, 450 μl of supernatant was mixed in 5-mm rounded-bottom NMR tubes with 50 μl D_2_O, and trimethylsilyl propionate (TSP) was added to reach a final concentration of 200 μM for use as a 0-ppm calibration standard. Data were collected using an 800 MHz Bruker Avance NMR spectrometer with a z gradient coil at 25°C. One-dimensional ^1^H-NMR analysis was performed with water suppression via presaturation or excitation sculpting to reveal peaks hidden by the water resonance (∼4.8 ppm). The recycle delay used to enable accurate integration of the peaks was 10 s.

### Detection of reactive oxygen species *in vivo*.

The cell-permeant dye 2′,7′-dihydrodichlorofluorescein diacetate (H_2_DCFDA) was used to detect endogenous ROS production in cell suspensions as described previously (77). Three independent E. coli cultures were grown to an OD_600_ of 1.0 in LB and were then washed and resuspended in phosphate-buffered saline (pH 7.4) to reach a final OD_600_ of 0.5. Cell suspensions were incubated at 37°C with 0.5 mM vanillin, vanillic acid, or vanillyl alcohol or with 0.1% (vol/vol) DMSO in the presence of 10 μM H_2_DCFDA. Fluorescence was measured in a Cary Eclipse fluorimeter with excitation at 485 nm and emission at 538 nm. Cell-free and dye-free controls showed no artefactual effects of the aromatic compounds on the measured fluorescence.

### Inductively coupled plasma mass spectrometry (ICP-MS).

E. coli cultures were grown in triplicate to an OD_600_ of 0.5 and centrifuged at 10,000 × *g* at 10°C for 20 min. Pellets were resuspended in wash buffer (10 mM HEPES, 0.5 M sorbitol, 100 μM EDTA, pH 7.5) and centrifuged and resuspended in fresh wash buffer twice more. Cells were finally washed once in wash buffer without EDTA. The pellets were resuspended in 1 ml 65% (vol/vol) nitric acid and left at room temperature for 48 h. The relative abundances of selected metals were determined using ICP-MS and an Agilent 4500 machine (Department of Chemistry, The University of Sheffield). The protein concentration of intact cells was determined using the Lowry method.

### Metal reduction assays.

Copper reduction was assayed *in vitro* at 37°C by addition of vanillin or related compounds to a reaction mixture containing 0.25 mM CuCl_2_ and 0.3 mM bathocuproinedisulfonic acid disodium salt (BCS) and HEPES (pH 7.4) buffer as described previously by Karlíčková et al. (78). The change in absorbance was measured at 484 nm. Control experiments were performed using DMSO as a nonreducing compound and with cuprous ions replacing CuCl_2_.

### DNA manipulation.

Standard cloning and transformation methods were used. Genomic DNA was extracted using a GenElute bacterial genome DNA kit (Sigma-Aldrich). PCR was performed using Phusion Flash High-Fidelity PCR master mix (Thermo Fisher Scientific) or KOD polymerase (Novagen), with products purified by the use of a QIAquick PCR purification kit (Qiagen). Restriction endonucleases, phosphatase, and ligases were purchased from New England Biolabs.

### Construction of plasmids and strains for endogenous vanillin production.

Fragments were generated for HIFI assembly (New England Biolabs) by PCR using the primers listed in Table S1. HIFI assembly was carried out as described by the manufacturer’s protocol. Initially, plasmid pTRC-*A. echfcs* was assembled using pTRC_HIFI_F and pTRC_HIFI_R primers for the vector backbone and codon-optimized, commercially synthesized *ech* and *fcs* genes from *Amycolatopsis* strain HR104 (Eurofins, Germany) containing 20-bp 5′ and 3′ overhangs complementary to pTRC. pTRC-*A.echfcs* was used for subsequent PCR amplification of *ech* and *fcs* for cloning into pSTV and pBAD vectors. The three plasmids were initially transformed into NEB5α cells, then each was transformed into DH5α, BL21(DE3), and BW25113 cells for expression studies. Genomic DNA of Pseudomonas putida S12 was used as a template to amplify the *ech* and *fcs* genes, employing primers (S12ech_BAD_F/R and S12fcs_BAD_F/R; see Table S1) that created an in-frame, 300-bp deletion in the vanillin dehydrogenase gene (located between *ech* and *fcs* in the P. putida S12 genome) in the assembled product. The genes were cloned by HIFI assembly into pBAD, and the construct was transformed into the E. coli strains described above.

### Protein overproduction and purification.

Overexpression constructs encoding C-terminal His tags were assembled for *yqhD*, *dkgA*, *yhbW*, and *gltA* by cloning amplicons derived from PCR using wild-type BW25113 DNA and the primer pairs shown in Table S1 (XXX_OE_F and XXX_OE_R) into pET-21a. Correct insertions were confirmed by sequencing with T7 primers and the plasmids transformed in E. coli BL21(DE3). Variant GltA enzymes with mutations matching those in strains EVT1 to EVT4 (see below) were produced by using primers gltA_OE_F and gltA_OE_R (Table S1) with genomic DNA from strains EVT1 to EVT4 as the templates to amplify fragments for cloning into pET21a as described above. The correct mutations were confirmed by automated DNA sequencing using primers gltA_sequence_F and gltA_sequence_R (Table S1). Overnight cultures of transformed E. coli were used to inoculate 1-liter volumes of LB in 2-liter flasks and were grown to an OD_600_ of ∼0.4 at 37°C, at which point expression was induced with 0.4 mM i9sopropyl β-d-1-thiogalactopyranoside (IPTG). Protein production typically lasted 3 h before cells were centrifuged (10,000 × *g* at 4°C, 10 min) and resuspended in binding buffer (20 mM sodium phosphate buffer, 0.5 M NaCl, 40 mM imidazole, pH 7.4). Cell-free extracts were made using a French press, and subsequent centrifugation (20,000 × *g* at 4°C for 20 min) was performed with proteins purified from the supernatants via nickel affinity chromatography using a HisTrap HP column (GE Healthcare). Concentration, desalting, and buffer exchange (where necessary) were conducted using Vivaspin columns (GE Healthcare) with appropriate MW cutoff values according to the manufacturer’s instructions. Protein concentrations were measured using the specific extinction coefficient calculated from the amino acid sequence and absorbance at 280 nm.

### Enzyme assays.

For fumarase assays, overnight cultures of E. coli were used to inoculate fresh LB to reach an OD_600_ of 0.05. Cells were grown to an OD_600_ of 0.5 and harvested by centrifugation (10,000 × *g* at 4°C for 10 min), and the pellets were resuspended in 50 mM Tris-HCl (pH 7.5). Cell-free extracts were prepared by sonication on ice and subsequent centrifugation to remove debris (20,000 × *g* at 4°C for 20 min). Total levels of fumarase activity in cell extracts were determined by measuring the rate of conversion of fumarate to malate. Cell-free extract (50 μl) was added to 50 mM Tris-HCl (pH 7.5) in a total volume of 1 ml, sodium fumarate was added to reach a final concentration of 0.4 mM to start the reaction, and the absorbance change was measured at 240 nm. Activity was determined using an extinction coefficient of 2.5 mM^−1 ^cm^−1^ at 240 nm, and the total protein concentration of cell extracts was determined using Bio-Rad protein assay dye reagent according to the manufacturer’s instructions. Purified YqhD and DkgA were assayed spectrophotometrically for vanillin reductase activity as described previously (21). Assay mixtures contained 100 mM phosphate buffer (pH 7.4) and 0.1 mM NADPH and a range of vanillin concentrations. Vanillin-dependent NADPH oxidation was measured at 340 nm at 37°C (ɛ_340_ = 6.2 × 10^3^ M^−1 ^cm^−1^), corrected for the decrease in vanillin absorbance at 340 nm (ɛ_340_ = 10.6 × 10^3^ M^−1 ^cm^−1^). Purified recombinant YhbW was assayed to determine whether it exhibited flavin-dependent monooxygenase activity with vanillin as the substrate. FMN was subjected to anaerobic photoreduction at a concentration of 25 μM in the presence of 0.5 μM proflavine, 5 mM EDTA, and 20 mM Tris-HCl (pH 7.4). A stock solution of vanillin was sparged with nitrogen for addition to the FMNH_2_ mixture. Assays were conducted anaerobically at 37°C with absorbance measured at 446 nm and initiated by the addition of aerobic enzyme solution or O_2_-saturated buffer. The rate of the increase of absorbance in the absence of vanillin was used as the baseline. Citrate synthase activity of cell extracts and purified enzymes was measured spectrophotometrically at 412 nm at 37°C by monitoring the reaction of coenzyme A with 5,5′-dithiobis(2-nitrobenzoic acid) (DTNB; ɛ_412_ = 13.6 × 10^3^ M^−1 ^cm^−1^). Typically, assays were conducted in 100 mM Tris-HCl buffer (pH 8), 0.2 mM DTNB, and 67 μM acetyl-CoA and were initiated by addition of oxaloacetate in a range of concentrations. Controls containing no protein were conducted, and a baseline rate before addition of oxaloacetate was recorded and subtracted to produce a corrected absorbance change value. All enzyme kinetic analyses were performed using GraphPad Prism v7.04 software. Propagation-of-error calculations for *k*_cat_/*K_m_* values were performed using the jacknife method (79), which takes into account the fact that *k*_cat_ and *K_m_* are not independent.

### Isolation and genome analysis of evolved vanillin tolerance (EVT) E. coli strains.

E. coli BW25113 cells were initially grown in 50 ml LB in 250-ml conical flasks containing 10 mM vanillin overnight before repeated daily subculturing to an initial starting OD_600_ of 0.02 into fresh medium containing vanillin over a 12-week period; the vanillin concentration used was gradually increased over this time to 20 mM. Four independent lines of subculturing in the presence of vanillin were maintained. Each transfer involved inoculation into three vanillin concentrations, with the culture with the highest concentration that exhibited growth used for the subsequent passage. Each line underwent an estimated 380 and 460 exponential-phase generations over the course of the experiment, with variations between lines occurring due to lack of growth during certain transfers. As a control, the parental E. coli cells were also subcultured in the absence of vanillin to provide a reference strain for genome sequencing. Single colonies of each of these five strains were isolated and replated, and samples were sent for whole-genome sequencing, conducted by MicrobesNG (Birmingham University, United Kingdom) using the Illumina platform. Single nucleotide polymorphisms (SNPs) and small insertions/deletions (indels) were identified using Snippy pipeline version 3.0 (80). The trimmed reads obtained from MicrobesNG were mapped to the genome of E. coli strain BW25113 (GenBank accession number CP009273) using BWA mem version 0.7.12 (81), and SNPs were called using FreeBayes version 1.1.0 (82). The default Snippy settings were used; those settings exclude SNPs identified in regions with less than 10× sequence coverage and SNPs supported by less than 90% of the overlapping reads. Larger structural variants (SVs) such as insertions, deletions, inversions, and other rearrangements were identified using LUMPY version 0.2.13 (83). SVs with fewer than 10 supporting pieces of evidence (split reads or discordant read pairs) were excluded from further analysis. The effects of the SNPs and structure variations (SVs) on any overlapping genes were determined using SnpEff (84). SNPs and SVs were displayed using the Dalliance Web browser (85).

### Data availability.

The mass spectrometry-based proteomics data have been deposited in the ProteomeXchange Consortium via the PRIDE partner repository (86) with data set identifier PXD009242. The genome sequence data have been submitted to the European Nucleotide Archive (ENA) with accession code PRJEB25786.
