# Supplementary figures and images for: Proteomic Profiling, Transcription Factor Modeling, and Genomics of Evolved Tolerant Strains Elucidate Mechanisms of Vanillin Toxicity in Escherichia coli
Source: mSystems. 2019 Jun 11;4(4):e00163-19. doi: 10.1128/mSystems.00163-19 (PMC6561319; doi:10.1128/mSystems.00163-19)

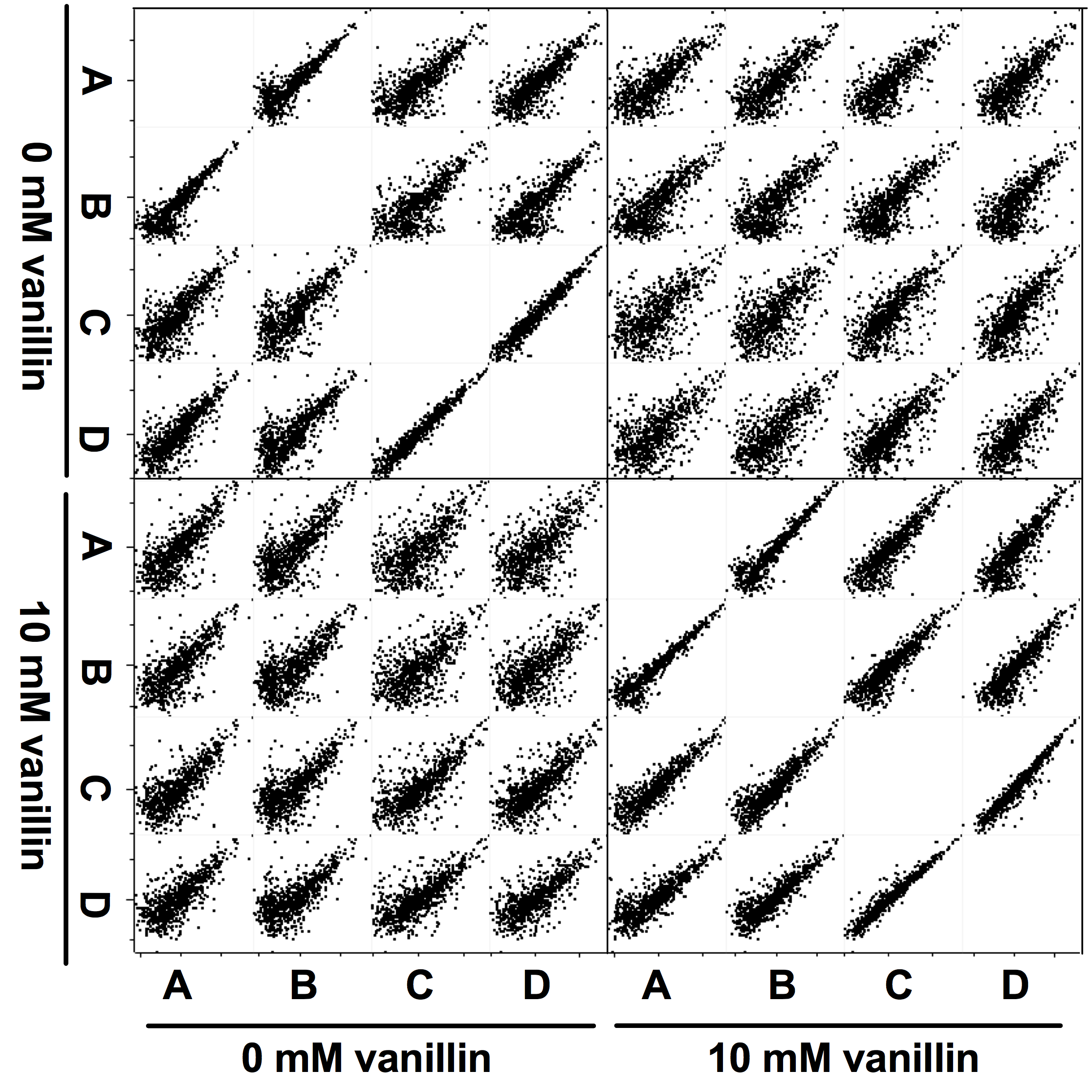

Supplement: FIG S1 [file mSystems.00163-19-sf001.tif]

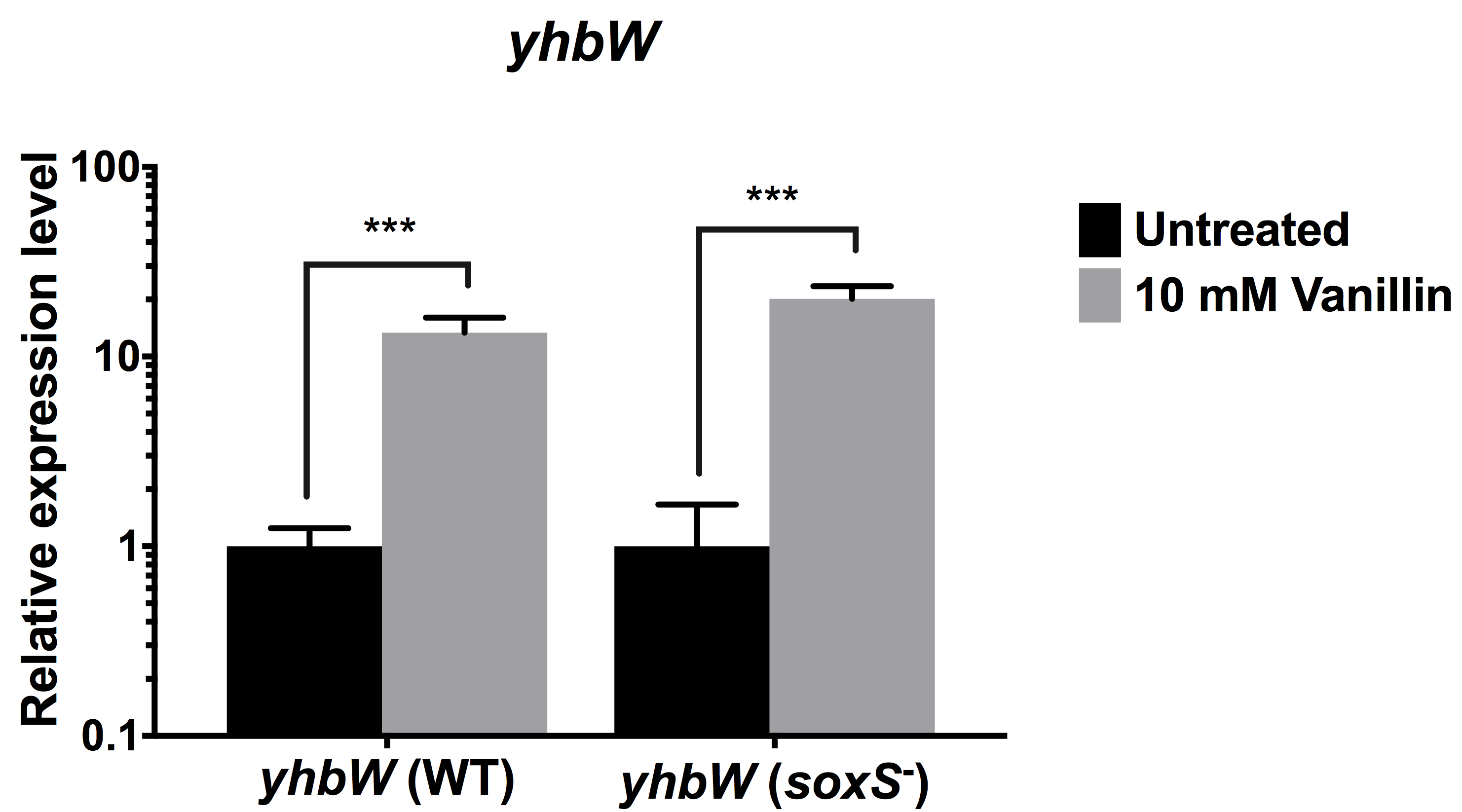

Supplement: FIG S2 [file mSystems.00163-19-sf002.tif]

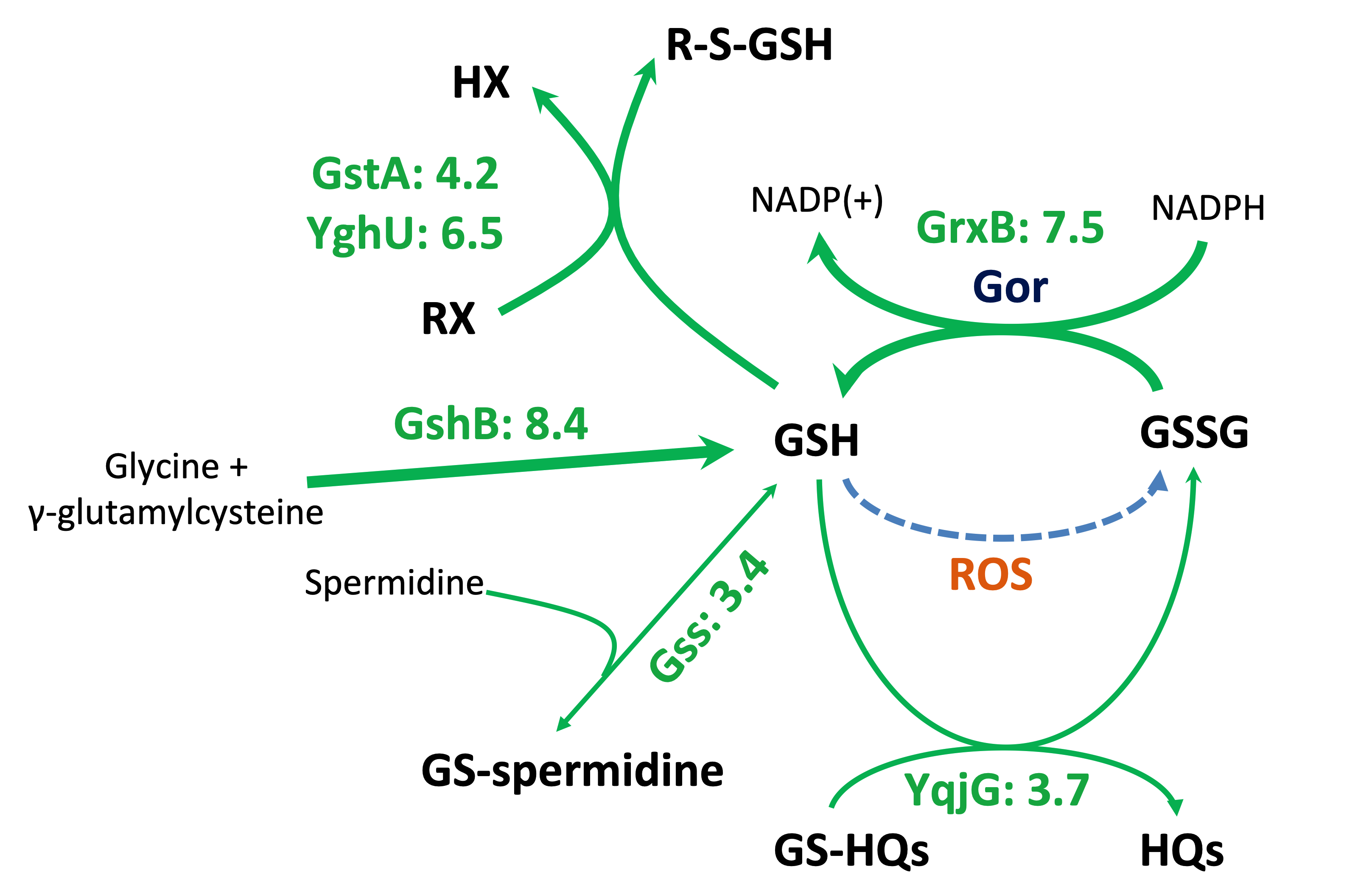

Supplement: FIG S3 [file mSystems.00163-19-sf003.tif]

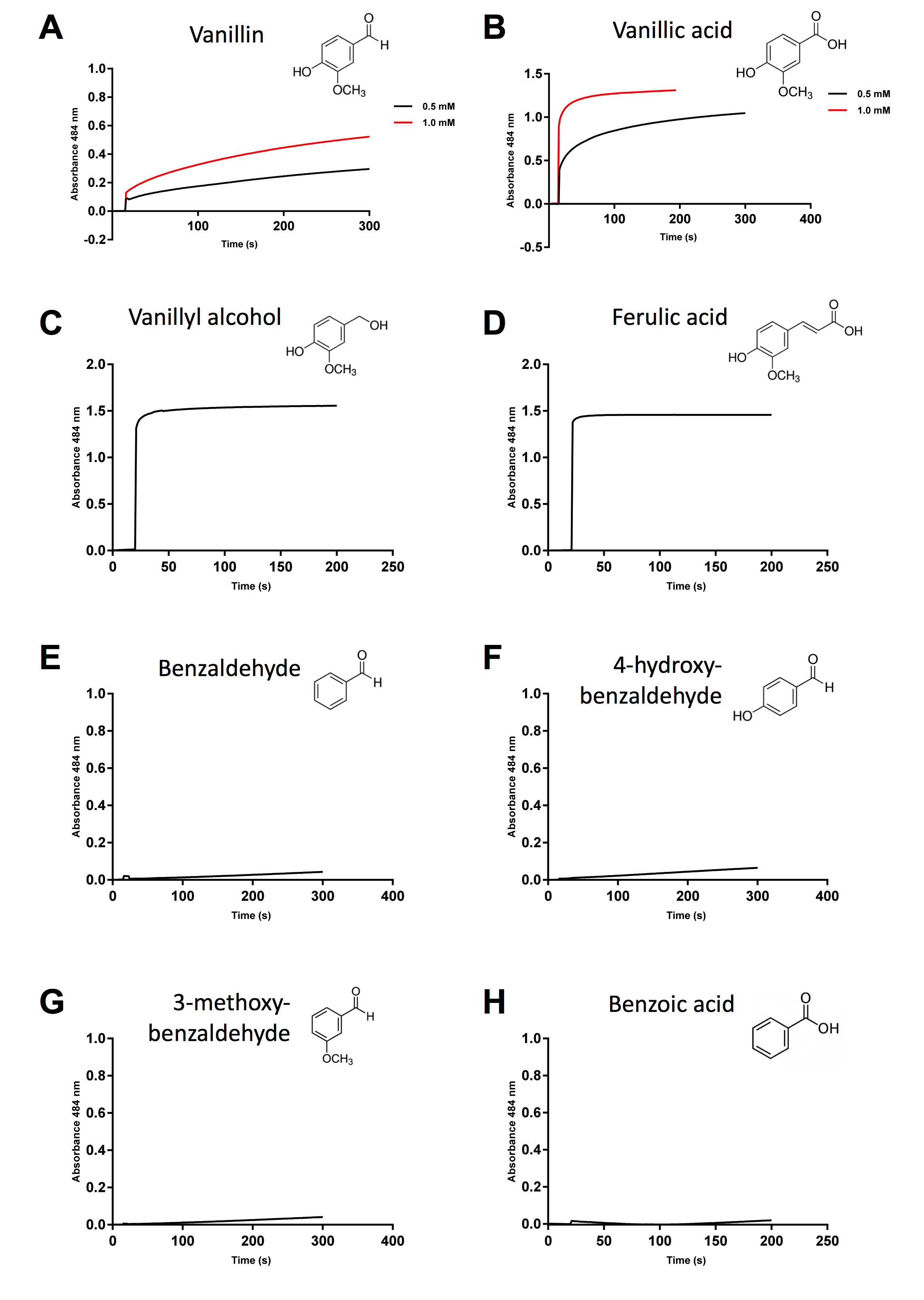

Supplement: FIG S4 [file mSystems.00163-19-sf004.tif]

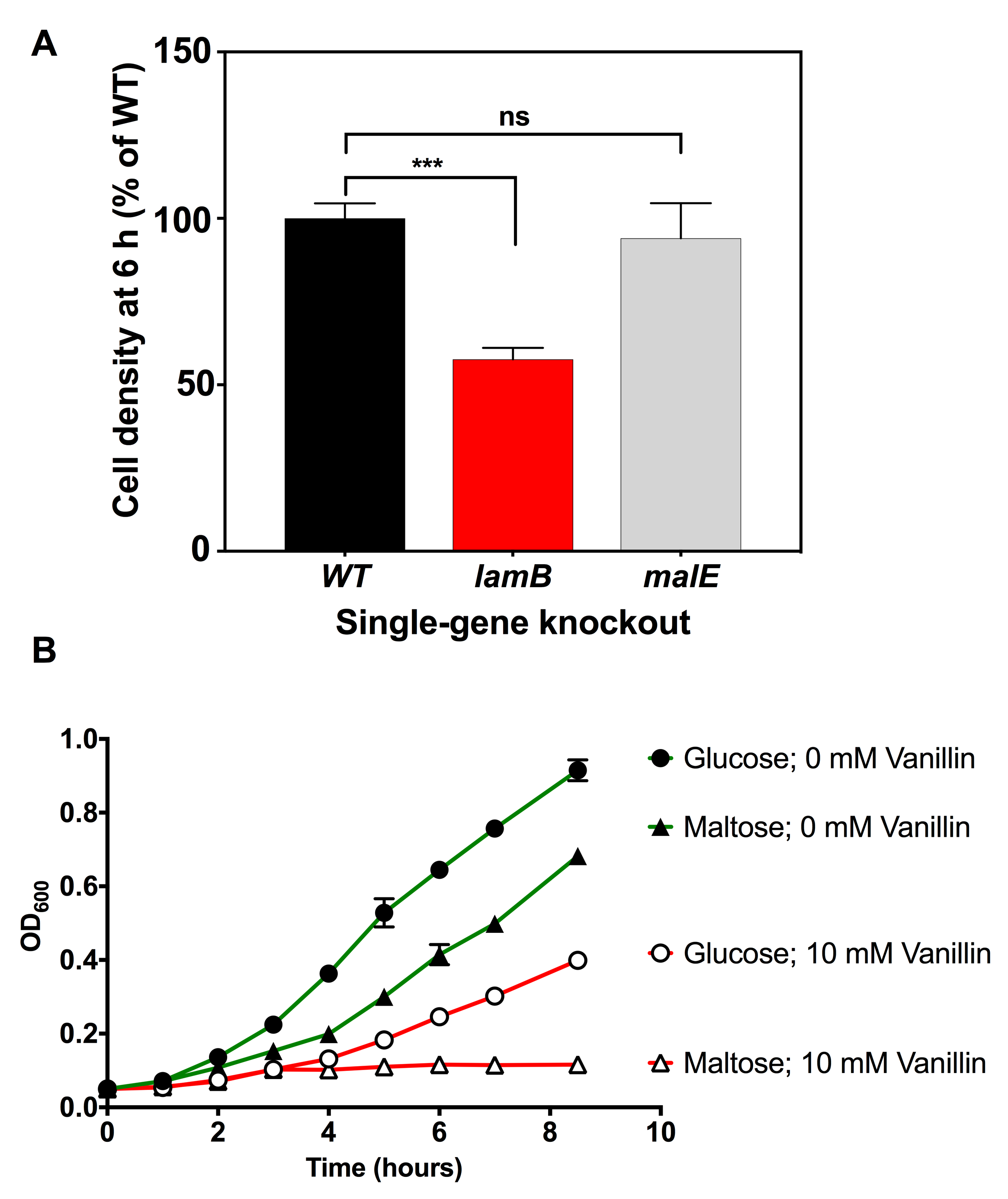

Supplement: FIG S5 [file mSystems.00163-19-sf005.tif]

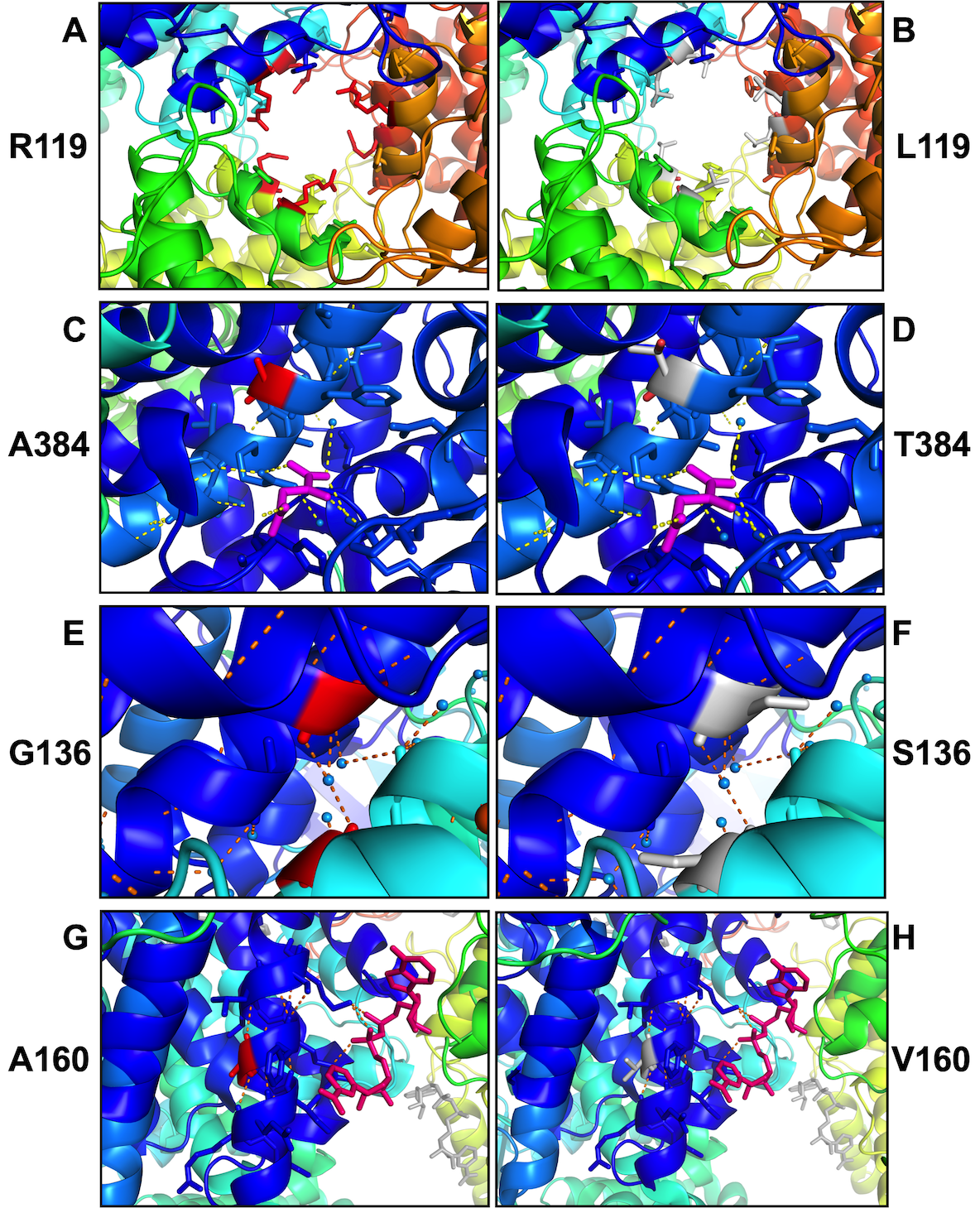

Supplement: FIG S6 [file mSystems.00163-19-sf006.tif]
