# Supplementary material for: Proteomic Profiling, Transcription Factor Modeling, and Genomics of Evolved Tolerant Strains Elucidate Mechanisms of Vanillin Toxicity in Escherichia coli
Source: mSystems. 2019 Jun 11;4(4):e00163-19. doi: 10.1128/mSystems.00163-19 (PMC6561319; doi:10.1128/mSystems.00163-19)
Supplement: TABLE S1 [file mSystems.00163-19-st001.pdf]

Table S1. Primers used in this study.

Primer	Sequence 5' → 3'
aaeA_RTPCR_F	GATGGTGAACAGTATCTGTCCT
aaeA_RTPCR_R	GTCGTATTAGTCATTCTGGCC
aaeB_RTPCR_F	GCAATACAGCCAATAAATGTG
aaeB_RTPCR_R	TGCTAACCAACATATTCGCT
acrA_RTPCR_F	TAGTAACAGTCAAAACTGAACC
acrA_RTPCR_R	TTTCGCACTGTCGTATGT
acrB_RTPCR_F	TAATTTCTTTATCGATCGCC
acrB_RTPCR_R	GTCACTGTTAGAGGACATGTA
acrD_RTCPR_F	TCTTTATTGATCGCCCCATT
acrD_RTPCR_R	TATCGAGGCCGGTCATATTT
acrF_RTPCR_F	TATTCGACGACCGATATTTGC
acrF_RTPCR_R	TGTTCGATAACCTGCGTCAC
copA_RTPCR_F	AAACTATCGACCTGACCCT
copA_RTPCR_R	TTTGGGTGGCTTACAGAT
dkgA_OE_F	CTTACATATGGCTAATCCAACCGTTATTAA
dkgA_OE_R	CTTACTCGAGGCCGCCGAACTGGTCA
gltA_OE_F	ATTGCATATGGCTGATACAAAAGCAAAACTCA
gltA_OE_R	ATTGCTCGAGACGCTTGATATCGCTTTTAAAG
gltA_sequence_F	ATTTTCACAATGGCACGT
gltA_sequence_R	GACGGTGAACATGGAAGA
marA_RTPCR_F	CAATACTGACGCTATTACCA
marA_RTPCR_R	GGTTTCTTTTTTAAACATCC
rrsA_RTPCR_F	ACCAGGGTATCAATCCTGTT
rrsA_RTPCR_R	GTTAATACCTTTGCTCATTGA
soxS_RTPCR_F	GCATATTGACCAGCCGCTTAA
soxS_RTPCR_R	TTACAGGCGGTGGCGATAATCGCT
yhbW_OE_F	ATTATGCTAGCACTGATAAAACCATTGCGTTTT
yhbW_OE_R	ATTATCTCGAGTCCCAACAACTCTTCCTTAAC
yhbW_RTPCR_F	GGACTGATAAAACCATTGCGT
yhbW_RTPCR_R	AGATGCAGCGTGGTGGTAT
yqhD_OE_F	ATATTAGCTAGCAACAACTTTAATCTGCACACCCCA
yqhD_OE_R	ATATTACTCGAGGCGGGCGGCTTCGTATAT
pTRC_HIFI_F	AAGCTTGGCTGTTTTGGCG
pTRC_HIFI_R	GGTCTGTTTCCTGTGTGAAATTG
pSTV_HIFI_F	GCATGCAAGCTTGGCACTG
pSTV_HIFI_R	TATTTATCCCCTTGAATTCGTAATCATGG
pBAD_HIFI_F	AGCTTGGCTGTTTTGGCGG
pBAD_HIFI_R	GGTGAATTCCTCCTGCTAG
fcsech_STV_F	CGAATTCAAGGGGATAAATAATGCGTAACCAAGGGCTGGG
fcsech_STV_R	CCAGTGCCAAGCTTGCATGCCTACTTTTCAGGATCGAATGCAG
fcsech_BAD_F	GCTAGCAGGAGGAATTCACCATGCGTAACCAAGGGCTGGG
fcsech_BAD_R	TCCGCCAAAACAGCCAAGCTCTACTTTTCAGGATCGAATGCAG
S12ech_BAD_F	GCTAGCAGGAGGAATTCACCATGAGCAAATATGAAGGCCGC
S12ech_BAD_R	TTGAGGTGGCGGGCCGCCAGGCGCAGGGCCATTGCGAAGC
S12fcs_BAD_F	CTGGCGGCCCGCCACCTCAA
S12fcs_BAD_R	TCCGCCAAAACAGCCAAGCTTCAAGGCCGCACCTTGGCGT
